# Ultra-small hollow ternary alloy nanoparticles for efficient hydrogen evolution reaction

**DOI:** 10.1093/nsr/nwaa204

**Published:** 2020-08-28

**Authors:** Zhenxing Li, Chengcheng Yu, Yikun Kang, Xin Zhang, Yangyang Wen, Zhao-Kui Wang, Chang Ma, Cong Wang, Kaiwen Wang, Xianlin Qu, Miao He, Ya-Wen Zhang, Weiyu Song

**Affiliations:** State Key Laboratory of Heavy Oil Processing, College of New Energy and Materials, Beijing Key Laboratory of Biogas Upgrading Utilization, China University of Petroleum (Beijing), Beijing 102249, China; State Key Laboratory of Heavy Oil Processing, College of New Energy and Materials, Beijing Key Laboratory of Biogas Upgrading Utilization, China University of Petroleum (Beijing), Beijing 102249, China; College of Science, China University of Petroleum (Beijing), Beijing 102249, China; State Key Laboratory of Heavy Oil Processing, College of New Energy and Materials, Beijing Key Laboratory of Biogas Upgrading Utilization, China University of Petroleum (Beijing), Beijing 102249, China; State Key Laboratory of Heavy Oil Processing, College of New Energy and Materials, Beijing Key Laboratory of Biogas Upgrading Utilization, China University of Petroleum (Beijing), Beijing 102249, China; Jiangsu Key Laboratory for Carbon-Based Functional Materials and Devices, Institute of Functional Nano and Soft Materials (FUNSOM), Soochow University, Suzhou 215123, China; State Key Laboratory of Heavy Oil Processing, College of New Energy and Materials, Beijing Key Laboratory of Biogas Upgrading Utilization, China University of Petroleum (Beijing), Beijing 102249, China; Beijing Key Laboratory and Institute of Microstructure and Property of Advanced Materials, Beijing University of Technology, Beijing 100124, China; Beijing Key Laboratory and Institute of Microstructure and Property of Advanced Materials, Beijing University of Technology, Beijing 100124, China; Beijing Key Laboratory and Institute of Microstructure and Property of Advanced Materials, Beijing University of Technology, Beijing 100124, China; State Key Laboratory of Heavy Oil Processing, College of New Energy and Materials, Beijing Key Laboratory of Biogas Upgrading Utilization, China University of Petroleum (Beijing), Beijing 102249, China; Beijing National Laboratory for Molecular Sciences, State Key Laboratory of Rare Earth Materials Chemistry and Applications, PKU–HKU Joint Laboratory in Rare Earth Materials and Bioinorganic Chemistry, College of Chemistry and Molecular Engineering, Peking University, Beijing 100871, China; College of Science, China University of Petroleum (Beijing), Beijing 102249, China

**Keywords:** ultra-small, hollow nanostructure, hydrogen evolution reaction, density functional theory

## Abstract

Hollow nanoparticles with large specific surface area and high atom utilization are promising catalysts for the hydrogen evolution reaction (HER). We describe herein the design and synthesis of a series of ultra-small hollow ternary alloy nanostructures using a simple one-pot strategy. The same technique was demonstrated for hollow PtNiCu nanoparticles, hollow PtCoCu nanoparticles and hollow CuNiCo nanoparticles. During synthesis, the displacement reaction and oxidative etching played important roles in the formation of hollow structures. Moreover, our hollow PtNiCu and PtCoCu nanoparticles were single crystalline, with an average diameter of 5 nm. Impressively, ultra-small hollow PtNiCu nanoparticles, containing only 10% Pt, exhibited greater electrocatalytic HER activity and stability than a commercial Pt/C catalyst. The overpotential of hollow PtNiCu nanoparticles at 10 mA cm^−2^ was 28 mV versus reversible hydrogen electrode (RHE). The mass activity was 4.54 A mg_Pt_^−1^ at −70 mV versus RHE, which is 5.62-fold greater than that of a commercial Pt/C system (0.81 A mg_Pt_^−1^). Through analyses of bonding and antibonding orbital filling, density functional theory calculations demonstrated that the bonding strength of different metals to the hydrogen intermediate (H^*^) was in the order of Pt > Co > Ni > Cu. The excellent HER performance of our hollow PtNiCu nanoparticles derives from moderately synergistic interactions between the three metals and H^*^. This work demonstrates a new strategy for the design of low-cost and high-activity HER catalysts.

## Teaser

The ultra-small hollow ternary alloy nanoparticles are synthesized via a simple one-pot method, and the PtNiCu nanoparticles exhibited excellent catalytic activity and stability in the hydrogen evolution reaction.

## INTRODUCTION

Ever-increasing energy consumption and growing environmental pollution have necessitated the development of new energy sources to replace fossil fuels [1–3]. At present, hydrogen (H_2_), a high-energy fuel that is environmentally friendly and easy to store, is considered to be the most promising clean energy source [4,5]. One particularly promising means of producing H_2_ is electrochemical water splitting [6,7]. The hydrogen evolution reaction (HER), a half-reaction of electrochemical water splitting, is an effective method for harvesting electrical energy from chemical energy [8]. Presently, the best solid catalyst for electrocatalytic H_2_ evolution is the noble metal platinum (Pt) [9–12]. However, Pt is relatively rare, rendering it expensive and not suitable for large-scale production [1,13]. Therefore, the design of less expensive catalysts with higher activity is paramount for large-scale application of the electrochemical HER. To reduce the cost of Pt-based electrocatalysts, it is necessary to ensure that the Pt atoms have a high utilization efficiency [14]. One way of doing this is to increase the degree of surface atom exposure by reducing the size of catalyst particles [15–21]. Although this strategy has been used widely, the relationship between electrocatalytic performance and the surface structure of catalyst particles is not entirely clear [14].

The preparation of nanomaterials with a specific morphology and/or size is important for practical application to nanoscience [22,23]. Nanomaterials featuring hollow structures have attracted great interest in recent years, since they are particularly applicable to electrocatalytic systems [14,24]. Hollow nanostructures containing noble metals and a highly open structure can greatly enhance the utilization efficiency of an electrocatalyst. Compared to similarly sized solid structures, hollow structures allow for greater specific surface area and expose more active sites to the surrounding medium [25–28]. These factors can have an enormous impact on the efficiency of a catalytic reaction.

The synthesis of hollow nanostructures of various compositions using a template-based method has been widely reported [29–31]. Xia and coworkers [32] removed the cores from nanoparticles by wet etching to obtain nanocages and nanoframes. Many previous studies have relied on seed-mediated methods to synthesize hollow nanoparticles. In our synthetic process, the hollow nanoparticles were directly obtained through galvanic displacement and oxidative etching on the nanoparticle surface. Although many reports describe the use of Pt-based nanocatalysts for electrocatalytic H_2_ evolution, most of the catalyst particles in these systems were relatively large (>10 nm) and contained high proportions of Pt (>50%) with low Pt utilization [10–12]. The amount of Pt needed in a catalyst can be greatly reduced by increasing its utilization. This can be accomplished by preparing catalysts with extremely small particle sizes and hollow structures.

We describe herein the design and synthesis of a series of ultra-small, hollow, ternary alloy nanostructures (PtNiCu, PtCoCu and CuNiCo) using a simple one-pot strategy. The average size of our hollow PtNiCu and PtCoCu nanoparticles was 5 nm. The PtNiCu nanoparticles contained only 10% Pt but provided a wealth of active sites and exhibited excellent electrocatalytic activity and stability, due to their unique hollow structure and large specific surface area. In an alkaline solution, the overpotential of hollow PtNiCu nanoparticles at 10 mA cm^−2^ was 28 mV versus reversible hydrogen electrode (RHE) with a corresponding Tafel slope of 52.1 mV per decade, which was lower than those of commercial Pt/C and PtCu nanoparticles. The mass activity was 4.54 A mg_Pt_^−1^ at −70 mV versus RHE, which is 5.62-fold higher than that of commercial Pt/C (0.81 A mg_Pt_^−1^). Density functional theory (DFT) calculations showed that the Δ*G*_H^*^_ of Pt–Ni–Cu was much closer to zero compared with Pt–Cu, exhibiting excellent HER activity in an alkaline solution due to synergistic effects between the three metals.

## RESULTS AND DISCUSSION

**Figure 1.**
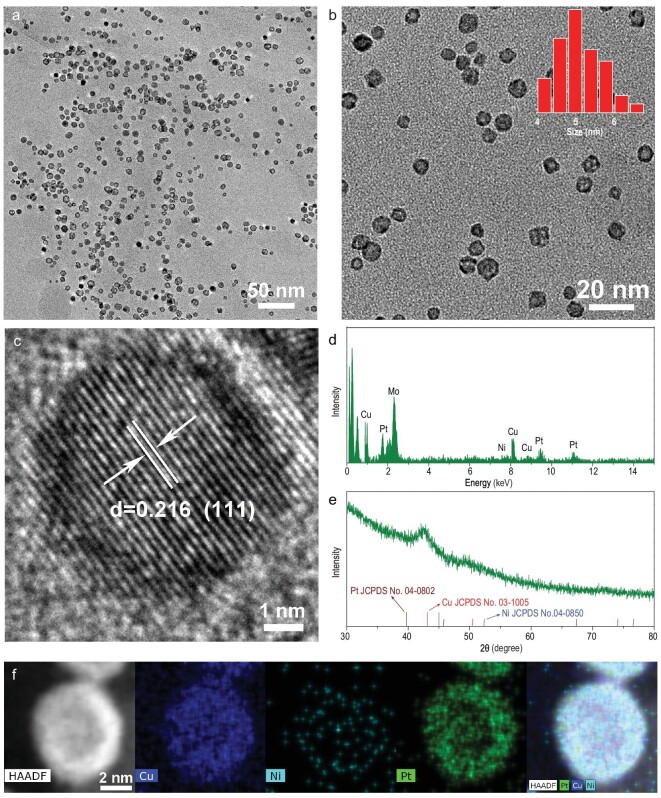
(a) TEM image of hollow PtNiCu nanoparticles. (b) High-magnification TEM image of hollow PtNiCu nanoparticles. Inset of panel (b) shows the size distribution of hollow PtNiCu nanoparticles. (c) HRTEM image of the hollow PtNiCu nanoparticle. (d) EDS of hollow PtNiCu nanoparticles. (e) XRD pattern of hollow PtNiCu nanoparticles. (f) EDX elemental mappings of the hollow PtNiCu nanoparticle.

In our nanoparticle synthesis, oleylamine (OAm) and ascorbic acid (AA) were used as the capping and reducing agents, respectively, and hollow PtNiCu nanoparticles were synthesized using a simple one-pot method. The morphology and structure of the as-synthesized hollow PtNiCu nanoparticles were analyzed by transmission electron microscopy (TEM) on carbon film. The TEM micrograph in Fig. 1 shows that the PtNiCu nanoparticles were uniformly dispersed without obvious agglomeration. Interestingly, the center of each PtNiCu nanoparticle was brighter than its edge, indicating a coreless shell. The high-angle annular dark field scanning TEM (HAADF-STEM) micrograph in Fig. S1 also indicates the existence of hollow structures, with a distinct contrast between the center and edge of each nanoparticle. The high-magnification micrograph in Fig. 1b shows that the hollow PtNiCu nanoparticles were monodisperse, with an average diameter of 5 nm. To the best of our knowledge, these are the smallest hollow nanoparticles to be reported [33]. The high-resolution TEM (HRTEM) micrograph in Fig. 1c indicates a lattice spacing of 0.216 nm, corresponding to the (111) crystal plane of hollow PtNiCu nanoparticles [34]. It is worth noting that the hollow PtNiCu nanoparticle was single crystal. By analysis of the fast Fourier transform (FFT) mode (see Fig. S2) of Fig. 1c, we can determine that the hollow PtNiCu nanoparticles are single crystals. TEM–energy-dispersive X-ray spectroscopy (EDX or EDS) (Fig. 1d) shows an elemental Pt : Ni : Cu ratio of 9 : 4 : 87, consistent with the results of inductively coupled plasma atomic emission spectroscopy (ICP-AES) (Pt : Ni : Cu = 10 : 4 : 86). Thus, the proportion of Pt in any given nanoparticle was as low as 10%. The X-ray diffraction (XRD) pattern of the prepared hollow PtNiCu nanoparticles in Fig. 1e shows a dominant peak at 42.49°, corresponding to the (111) facets, and two weaker peaks at 49.41° and 72.21°, corresponding to the (200) and (220) facets, respectively. These data are characteristic of a typical face-centered cubic (fcc) crystal. Note that each diffraction peak of the hollow PtNiCu nanoparticles was located between the corresponding peak positions of fcc Pt (JCPDS No. 04-0802), Cu (JCPDS No. 03-1005) or pure Ni (JCPDS No. 04-0850). Also note that peaks corresponding to pure phases of each of these elements were not observed. Therefore, our hollow PtNiCu nanoparticles feature an alloy phase structure. An HAADF-STEM image (see Fig. S3) and EDX mapping (Fig. 1f) show that the Pt, Ni and Cu elements are evenly distributed in hollow PtNiCu nanoparticles, which further confirmed the formation of PtNiCu alloy phase structure, agreeing with the result of XRD pattern. The EDX line scan (see Fig. S4) shows that the contents of Pt, Ni and Cu in the hollow nanoparticles are lower in the center, confirming the formation of the hollow structure.

**Figure 2.**
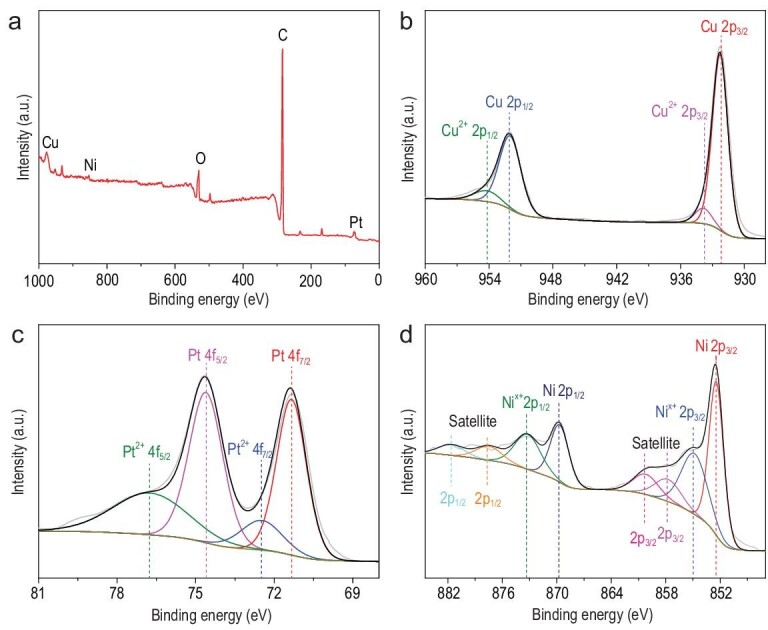
(a) XPS survey spectrum of the hollow PtNiCu nanoparticle. (b) Cu 2p XPS spectrum, (c) Pt 4f XPS spectrum and (d) Ni 2p XPS spectrum of the hollow PtNiCu nanoparticle.

The valence state of our hollow PtNiCu alloy nanoparticles was determined using X-ray photoelectron spectroscopy (XPS). The XPS survey spectrum in Fig. 2a contains clear peaks corresponding to Pt, Ni, Cu, O and C. The C and O signals were attributed to small amounts of residual organic material. The data in Table S2 indicate an atomic Pt : Ni : Cu ratio of 10 : 5 : 85, which closely matches the results of the EDX and ICP-AES analyses. Figure 2a clearly shows that the elements Pt, Ni, Cu, O and C are present in the XPS survey spectrum, and the signal appearance of C and O is attributed to background C element calibration and a small amount of organic remaining. As can be seen from Fig. 2b, the Cu 2p of the hollow PtNiCu nanoparticle had two peak regions, the binding energy of 952.1 eV assigned to Cu 2p_1/2_ and the binding energy of 932.3 eV assigned to Cu 2p_3/2_, corresponding to Cu(0). In addition, the binding energies of 954.2 and 933.8 eV can be attributed to the Cu^2+^ on the nanoparticles. Figure 2c shows that the Pt 4f region of the hollow PtNiCu nanoparticles can be divided into two pairs of peaks. The binding energies of Pt 4f peaks are 74.5 and 71.4 eV, corresponding to the Pt 4f_5/2_ and Pt 4f_7/2_ of Pt(0), respectively. The other peaks at 76.3 and 72.5 eV can be assigned to the Pt^2+^ species, which showed the presence of PtO or Pt(OH)_2_ in hollow PtNiCu nanoparticles. The XPS spectrum of Ni 2p is shown in Fig. 2d; the binding energies of Ni 2p_1/2_ and Ni 2p_3/2_ are 869.8 and 852.5 eV, respectively, and can be assigned to Ni(0). The binding energies of Ni*^x^*^+^ 2p_1/2_ and Ni*^x^*^+^ 2p_3/2_ are 869.8 and 852.5 eV, corresponding to satellite peaks observed at 881.7, 877.8, 860.4 and 857.9 eV, respectively, which confirmed the partial oxidation of Ni in the prepared sample.

Key experimental parameters were varied to better elucidate the growth mechanism of our hollow PtNiCu nanoparticles. The amount of metal precursor was a particularly important factor in the formation of uniform hollow nanoparticles. Reducing the amount of Cu(acac)_2_ to 5 mg yielded solid spherical particles of uneven size (see Fig. S5a). Increasing the amount of Cu(acac)_2_ to 10 mg resulted in hollow nanoparticles (see Fig. S5b), but the particle size was still heterogeneous. The amount of reducing agent AA and the presence of dissolved O_2_ were also crucial factors in the synthesis of hollow nanostructures. Reducing the amount of AA to 25 mg resulted primarily in nanospheres (see Fig. S6a), and increasing the amount of AA to 100 mg resulted in relatively large hollow nanospheres (see Fig. S6b). The reaction kinetics is affected by the concentration of the reducing agent. The higher the concentration and the stronger the driving force of the reaction, the larger the average size of the nanoparticles obtained [35]. The hollow nanostructure disappeared when the air in the reaction solution was removed with N_2_ (see Fig. S7). O_2_ in OAm can oxidize and etch Ni of nanoparticles [36]. Therefore, air is a vital factor to synthesize the unique hollow PtNiCu nanoparticles. In addition, the Brunauer–Emmett–Teller surface areas of hollow PtNiCu nanoparticles and PtNiCu nanoparticles are measured to be 34.54 and 14.42 m^2^ g^−1^, respectively, indicating that hollow PtNiCu nanoparticles show larger specific surface area than PtNiCu nanoparticles. The pore size distribution of hollow PtNiCu nanoparticles (see Fig. S8) is extremely narrow, and the pore size is 1.8 nm.

**Figure 3.**
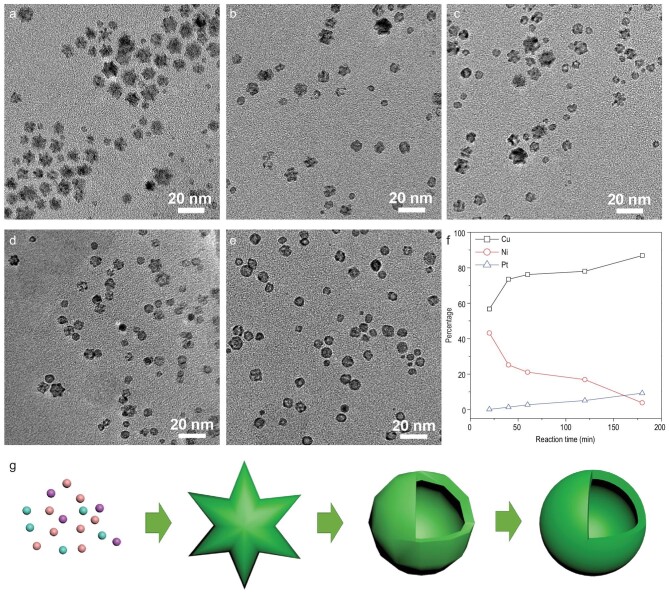
Representative TEM images of hollow PtNiCu nanoparticle intermediates collected at (a) 20 min, (b) 40 min, (c) 60 min, (d) 120 min and (e) 180 min. (f) The percentage of Pt, Ni and Cu for the hollow PtNiCu nanoparticle intermediates. (g) Synthetic diagram of hollow PtNiCu nanoparticles.

The growth and structural evolution of hollow PtNiCu nanoparticles were monitored by TEM (Fig. 3a–e) and EDS (Fig. 3f) analyses. After 20 min, a branched structure was obtained (Fig. 3a) consisting almost entirely of Ni and Cu. Almost no Pt (Fig. 3f) was present, although the standard redox potentials of Cu^2+^/Cu (0.34 V) and Ni^2+^/Ni (−0.25 V) are more negative than that of Pt^2+^/Pt (1.18 V). Thus, AA dissolved in OAm facilitated the preferential reduction of Cu^2+^ and Ni^2+^ ions [37–39]. After the growth reaction had proceeded for 40 min, the branched structures were further reduced and hollow nanoparticles began to appear (Fig. 3b). At this point, small amounts of Pt began to appear in the product (Fig. 3f). After 60 min, large amounts of hollow nanoparticles appeared (Fig. 3c), with continuously increasing percentages of Pt. At 120 min, most of the branched nanoparticles had been converted to hollow nanoparticles (Fig. 3d). After 180 min, all of the nanoparticles exhibited hollow structures (Fig. 3e). In addition, as the reaction proceeded, the percentages of Cu and Pt in the product increased continuously, while the percentage of Ni decreased (Fig. 3f). Therefore, both galvanic displacement and oxidative etching occurred on the surface of the branched nanoparticles. On the one hand, Pt^2+^ ions in the solution were reduced by Ni on the surface of the branched nanoparticles. On the other hand, the solution was acidic due to the presence of AA; a portion of the Ni in the branched particles was oxidatively etched [24]. This process was ultimately responsible for the decreasing percentage of Ni in the final nanoparticles. No significant changes were evident in the morphology or elemental composition (Pt : Ni : Cu = 9 : 4 : 87) of the nanoparticles between 180 and 240 min (see Fig. S9). Figure 3g shows a schematic diagram detailing the synthesis of hollow PtNiCu nanoparticles.

**Figure 4.**
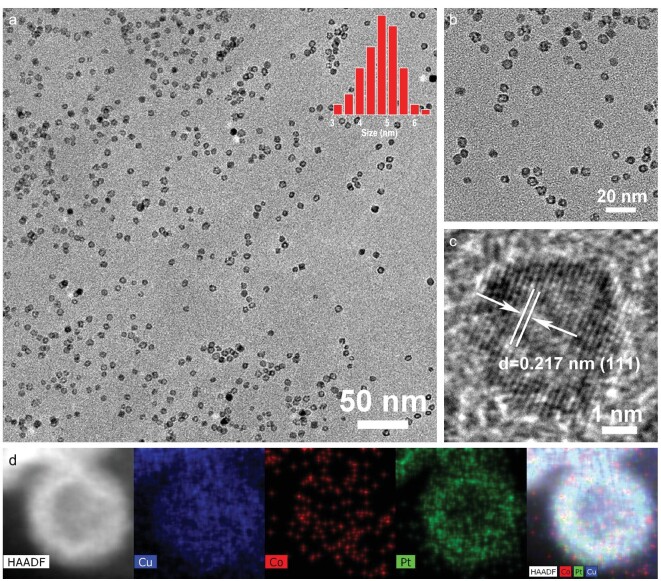
(a) TEM image of hollow PtCoCu nanoparticles. Inset of panel (a) shows the size distribution of hollow PtCoCu nanoparticles. (b) High-magnification TEM image of hollow PtCoCu nanoparticles. (c) HRTEM image of the hollow PtCoCu nanoparticle. (d) EDX elemental mappings of the hollow PtCoCu nanoparticle.

This synthetic strategy is generally suitable for obtaining hollow nanostructures from different materials, including PtCoCu and CuCoNi. The TEM micrographs in Fig. 4a and b show a uniformly shaped and monodisperse sample of hollow PtCoCu nanoparticles. The corresponding particle size distribution is shown in the inset of Fig. 4a. The diameter of these hollow PtCoCu nanoparticles ranged between 3.5 and 6.5 nm, with an average value of 5 nm. The HRTEM micrograph in Fig. 4c clearly shows a lattice spacing of 0.217 nm, corresponding to the (111) crystal plane of a hollow PtCoCu alloy nanoparticle [34]. The hollow PtCoCu alloy nanoparticle was also single crystal. FFT of the data in Fig. 4c shows that our hollow PtCoCu nanoparticles are single crystalline (see Fig. S10). The XRD peak positions of hollow PtCoCu were 41.6°, 48.3°

and 71.1°, respectively, and located between those of Pt (JCPDS No. 04-0802), Co (JCPDS No. 01-1225) and Cu (JCPDS No. 03-1005) along with a slightly negative shift (see Fig. S11). The position of the main peak in the XRD pattern was consistent with the lattice spacing and crystal plane of hollow PtCoCu nanoparticles, indicating the formation of a PtCoCu alloy. The elemental composition of Pt : Co : Cu was determined to be 7 : 11 : 82 by EDS (see Fig. S12) and the proportion of Pt was lower than that observed with hollow PtNiCu alloy nanoparticles. STEM-EDS elemental mapping (Fig. 4d) showed that Pt, Ni and Cu were uniformly dispersed throughout the hollow nanoparticles, providing further evidence for the successful synthesis of hollow PtCoCu alloy nanocrystals. XPS spectra (see Fig. S13 and Table S2) were used to characterize the surface composition and valence states of hollow PtCoCu nanoparticles. Pt, Co and Cu on the nanoparticle surface were easily oxidized in the measurement of XPS, and XPS spectrum shows that Pt, Co and Cu were all in partially oxidized states [40].

This same synthetic strategy was also effective in the synthesis of hollow CuCoNi nanoparticles. The TEM micrograph in Fig. S14 shows that the average edge length of a hollow CuCoNi nanoparticle was 22 nm. Since the bonding ability of Pt with surrounding metals is stronger than Cu, the total system can increase in the absence of Pt. In order to maintain the surface energy in this environment, the size of the nanoparticles becomes larger [41]. The high-magnification TEM micrograph in Fig. S15a clearly shows the hollow structure of our CuCoNi nanoparticles. EDS elemental mapping (see Fig. S15b) showed that Cu, Co and Ni were uniformly dispersed at the edge of the particle, confirming the formation of a hollow CuCoNi nanoalloy. The lattice spacing of hollow CuCoNi nanoparticle was 0.262 nm (see Fig. S16), corresponding to the EDS mapping [42].

**Figure 5.**
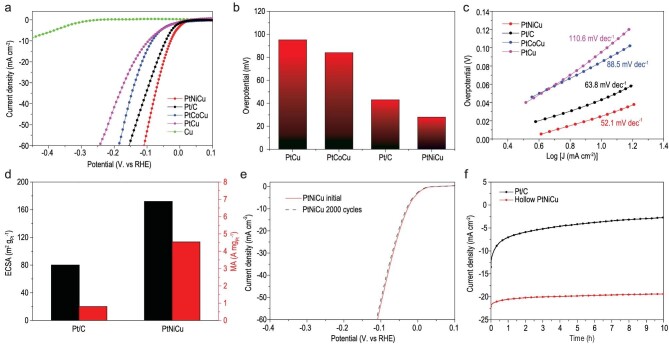
(a) The HER polarization curves of hollow PtNiCu nanoparticles, commercial Pt/C, hollow PtCoCu nanoparticles, hollow PtCu nanoparticles and Cu nanoparticles in 1.0 M KOH aqueous solution at a scan rate of 5 mV s^−1^. (b) Overpotentials at a current density of 10.0 mA cm^−2^ of hollow PtNiCu nanoparticles, commercial Pt/C, hollow PtCoCu nanoparticles and hollow PtCu nanoparticles. (c) Tafel plots of the hollow PtNiCu nanoparticles, commercial Pt/C, hollow PtCoCu nanoparticles and hollow PtCu nanoparticles. (d) ECSA (left axis) of hollow PtNiCu nanoparticles and commercial Pt/C; mass activity (right axis) for HER at −70 mV versus RHE. (e) Long-term cycling test of hollow PtNiCu nanoparticles. The polarization curves were recorded before and after 2000 cycles in 1.0 M KOH aqueous solution. (f) Current–time curves of hollow PtNiCu nanoparticles and commercial Pt/C in an N_2_-saturated 1.0 M KOH solution at the working potential of 50 mV versus RHE.

The water–alkali and chlor-alkali industries are the main ways of electrochemical hydrogen production [1,43,44]. However, the Pt catalyst generally has a lower HER activity in an alkaline solution than an acidic solution [43,44]. It is extremely meaningful to develop a catalyst with high activity and high Pt utilization in alkaline conditions. The electrochemical hydrogen evolution performance of catalysts in 1.0 M KOH electrolyte was evaluated in a three-electrode system. Figure 5a shows polarization curves of hollow PtNiCu, PtCoCu and PtCu nanoparticles (see Fig. S17) and solid Cu nanoparticles, determined by linear sweep voltammetry (LSV) at a scan rate of 5 mV s^−1^. For comparison, a 20% commercial Pt/C catalyst was evaluated under the same conditions. The column chart (Fig. 5b) clearly shows that the overpotentials of the as-prepared hollow PtNiCu nanoparticles, commercial Pt/C, hollow PtCoCu nanoparticles and hollow PtCu nanoparticles at a current density of 10 mA cm^−2^ are 28, 43, 84 and 95 mV versus RHE, respectively. Obviously, the hollow PtNiCu nanoparticles showed the lowest overpotential than commercial Pt/C, hollow PtCoCu nanoparticles and hollow PtCu nanoparticles, indicating that the hollow PtNiCu nanoparticles exhibited excellent HER catalytic activity. In addition, the polarization curves of hollow CuCoNi nanoparticles (see Fig. S18) were measured for HER performance.

The Tafel slope was related to the catalyst material and the surface state of the catalyst, and can reflect the electron transfer rate-limiting step in different catalyst systems [45,46]. The Tafel slope of our as-prepared hollow PtNiCu nanoparticles was 52.1 mV per decade, which is much smaller than those of Pt/C (63.8 mV per decade), hollow PtCoCu nanoparticles (88.5 mV per decade) and hollow PtCu nanoparticles (110.6 mV per decade) (Fig. 5c). In general, decorating Pt atoms with transition metals (Co, Ni) enhance their catalytic activity [47–50]. As can be seen from the above measurement results, the hollow PtNiCu nanoparticles and PtCoCu nanoparticles exhibit higher HER catalytic activity than hollow PtCu nanoparticles when a

transition metal Co or Ni is added to PtCu. In addition, the PtNiCu nanoparticles have a large specific surface area and can provide more active sites due to the unique hollow structure. Therefore, our hollow PtNiCu nanoparticles demonstrate greater HER catalytic performance than most Pt-based catalysts reported to date (see Table S7).

In order to evaluate the electrochemically active surface area (ECSA; see Fig. S19) of the hollow PtNiCu nanoparticles and Pt/C, the cyclic voltammetry (CV) curves were measured in N_2_-saturated 0.5 M H_2_SO_4_ with a scan rate of 50 mV s^−1^ [51]. Figure 5d shows that the ECSA values of the hollow PtNiCu nanoparticle and commercial Pt/C are calculated to be 81 and 169 m^2^ g_Pt_^−1^. The ECSA of our hollow PtNiCu nanoparticles was more than twice that of commercial Pt/C. This can be attributed to the hollow structure and relatively large number of active sites. In addition, the double-layer capacitance (*C*_dl_) values of hollow PtNiCu nanoparticles and Pt/C were calculated from the CV curves (see Fig. S20); the values were 4.04 and 2.79 mF cm^−2^, respectively (see Fig. S21), which were corresponding to the results of ECSA. Specifically, Fig. 5d shows that at an overpotential of 70 mV versus RHE, the mass activity of the hollow PtNiCu nanoparticles was 4.54 A mg_Pt_^−1^, which is 5.62-fold greater than that of commercial Pt/C (0.81 A mg_Pt_^−1^). In addition, the hollow PtNiCu nanoparticles were incredibly stable over long-term electrochemical cycling. The polarization curve of hollow PtNiCu nanoparticles exhibited almost no changes over 2000 cycles from 0.1 to −0.2 V versus RHE in an N_2_-saturated 1.0 M KOH solution (Fig. 5e). However, there was a significant shift in the polarization curve of commercial Pt/C after 1000 cycles (see Fig. S22). The current–time curves in Fig. 5f further demonstrate the electrochemical stability of our hollow PtNiCu nanoparticles. Almost no shift in current density was observed with hollow PtNiCu nanoparticles at 50 mV versus RHE after 10 h of durability testing. In contrast, the current density of the commercial Pt/C system dropped from 13.5 to 2.8 mA cm^−2^ over the same period. Especially, the PtNiCu nanoparticles after the electrocatalytic stability test were characterized by TEM (see Fig. S23), which clearly showed that the size and morphology of PtNiCu nanoparticles were still retained, demonstrating that the PtNiCu nanoparticles showed high stability. Electrochemical impedance spectra (EIS) were measured to determine the electron transfer efficiencies of these catalyst systems. The Nyquist semicircle of PtNiCu, shown in Fig. S24, was much smaller than those of commercial Pt/C and PtCu nanoparticles, indicating excellent electronic conductivity. These data are consistent with the results of LSV analyses.

The DFT calculations were performed to reveal the origin of the superior HER activities of the hollow PtNiCu nanoparticle. Generally, the adsorption free energy of hydrogen (Δ*G*_H^*^_) has been considered as a valid descriptor for HER catalytic activity [52]. The Δ*G*_H^*^_ values of nanoparticles with different compositions were calculated to explore rate-determining steps (RDSs) in the experiment. In view of the fact that H^*^ adsorbs on Cu (111) substrate at the hollow site, we simulated the alloys of different components by replacing the metal atoms that make up the hollow adsorption site. The theoretical models of Pt–Ni–Cu, Pt–Co–Cu, Pt–Cu and Cu are shown in Fig. S25, corresponding to the prepared hollow PtNiCu nanoparticle, hollow PtCoCu nanoparticle, hollow PtCu nanoparticle and Cu nanoparticle, respectively. In addition, the doping energy (Δ*E*_dop_) was calculated to determine the thermodynamically favorable configuration of the Pt–Cu model (see Fig. S26). Note that the adsorbed H^*^ interacts with three metal atoms at the hollow site (see Fig. S25f–i). |Δ*G*_H^*^_| was expected to approach zero for the optimal HER catalyst [52].

**Figure 6.**
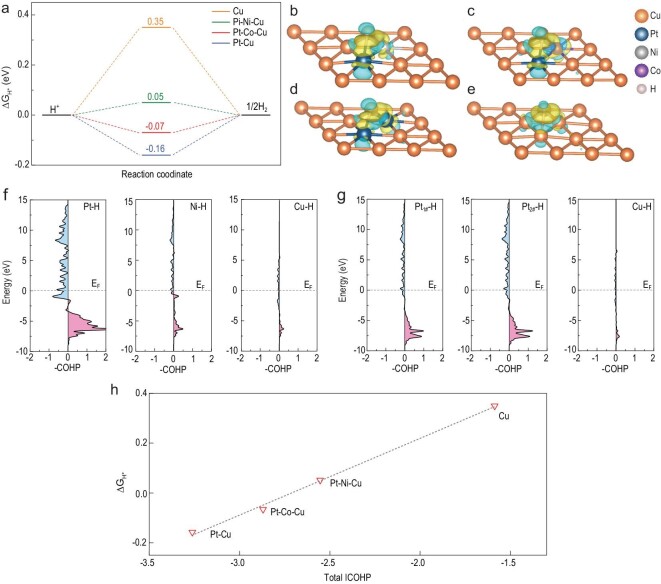
(a) Free energy diagram of HER at equilibrium potential for Pt–Ni–Cu, Pt–Co–Cu, Pt–Cu and Cu. The charge density difference of H^*^ adsorption on (b) Pt–Ni–Cu, (c) Pt–Co–Cu, (d) Pt–Cu and (e) Cu. Yellow (blue) isosurfaces denote an increase (decrease) of 0.01 e Å^−3^ for electronic density. pCOHP for (f) Pt–H, Ni–H and Cu–H on the Pt–Ni–Cu model at the hollow adsorption site, and for (g) Pt_1#_–H, Pt_2#_–H and Cu–H on the Pt–Cu model, respectively. (h) The correlation between the total ICOHP and Δ*G*_H^*^_.

The free energy diagrams in Fig. 6a show that the binding of H^*^ onto pure copper was weak (Δ*G*_H^*^_ = 0.35 eV), resulting in limited H^+^ reduction. Pt–Cu and H^*^ were so strongly adsorbed that the value of Δ*G*_H^*^_ was −0.16 eV, and the RDS was the desorption of H_2_, indicating the poor catalytic activity for HER. With the addition of Co or Ni during synthesis, the Gibbs free energy of adsorbed H^*^ on the catalyst surface changed greatly due to the activation of adjacent Pt atoms by Co or Ni atoms. Compared to the hollow PtCu nanoparticles, the Δ*G*_H^*^_ of the Pt–Ni–Cu was 0.05 eV and closer to zero, indicating the excellent HER activity due to the synergistic effect of three metals. The H^*^ adsorption of the Pt–Co–Cu was relatively stronger than that of Pt–Ni–Cu, resulting from the negative Δ*G*_H^*^_ (−0.07 eV). Moreover, we also calculated the free energy of the water dissociation process on the investigated models as shown in Fig. S27, and the ability of water dissociation is consistent with our conclusions using |Δ*G*_H^*^_| as a descriptor. The electronic structure of H^*^ adsorbed on a site inside a hollow nanoparticle can be preliminarily estimated based on charge density differences (Fig. 6b–e). Clearly, the localization of electrons around Pt–H, Ni–H and Co–H bonds indicated that Pt, Ni and Co atoms made contribution to adsorption, while the decrease of electrons between Cu–H bonds implied the weak binding of copper to H^*^. Therefore, the poor HER performance of pure Cu nanoparticle can be explained. The results of these theoretical calculations were consistent with the experimental data and confirm the feasibility of designing a high-performance HER catalyst using this simple synthetic strategy.

To further look into the origin of HER performance and the role of Pt, Ni and Co components, the projected crystal orbital Hamilton population (pCOHP) was calculated to reveal the interaction between the metal atoms and the hydrogen adatom [53]. Since H^*^ interacts with three metal atoms on the slab, we calculate each metal–H bond separately among the investigated models. (The pCOHP diagrams of each metal–H bond for Pt–Ni–Cu and Pt–Cu are shown in Fig. 6f and g, and for Pt–Co–Cu and Cu are shown in Fig. S28). In the pCOHP diagram, positive and negative values correspond to bonding and antibonding contributions, respectively. It was initially observed that antibonding orbits were partially filled when H^*^ was bonded to the metal atoms in a hollow site, indicated by the blue area below the Fermi level. In addition, contributions from bonding orbitals of Pt–H were large among these metal–H bonds, while those of Ni–H (Fig. 6f) and Co–H (see Fig. S28b) were relatively moderate, and that of Cu–H was miniscule. To better understand metal–H bond strength in these systems, we computed the integrated COHP (ICOHP) by calculating the energy integral up to the Fermi level for each metal–H bond, and summed the three ICOHP values for each hollow adsorption site to obtain a total ICOHP. The details of these calculations are provided in Tables S3–S6. Through the quantitative analysis, the ICOHP of Pt–H bond was relatively more negative (∼−1.60) while that of Cu–H was close to 0 (∼−0.40), indicating that Pt was the strongest combination with H^*^ among the investigated elements, and Cu was the weakest. This is consistent with the poor HER performance of Pt–Cu and Cu systems, which are limited by H_2_ desorption and H^+^ adsorption, respectively. This is further evidenced by the total ICOHP values of Pt–Cu and Cu (−3.26 and −1.59). The bonding ability of H^*^ to Ni and Co atoms was moderate, with ICOHP values of −0.74 and −1.12, respectively (see Tables S3 and S4). Thus, the transition metal played a key role in balancing and optimizing the adsorption capacity for H^*^. In addition, the performance of Pt–Co–Cu was slightly worse than that of Pt–Ni–Cu because the absorption of H^*^ by Co atom was slightly stronger than that of Ni, which led to the stronger comprehensive ability of H adsorption at the hollow site on Pt–Co–Cu. This will be further elaborated in the following. To reveal the origin of the difference of Δ*G*_H^*^_ among the investigated models, the linear relationship between the total ICOHP and Δ*G*_H^*^_ is shown in Fig. 6h. This linear correlation gave a quantitative explanation for the adsorption of H^*^ at hollow sites with different constituent elements and prediction of the catalyst design. With the increase of the total ICOHP, Δ*G*_H^*^_ changed from negative to positive, indicating the decreasing binding strength of H^*^ at hollow site. Based on the above discussion, the adsorption strength of metal atoms for H^*^ was Pt > Co > Ni > Cu. In the design of HER catalyst, the energetic descriptor, Δ*G*_H^*^_, was routinely employed, which was assumed to reach zero for the optimal HER catalyst. The linear relationship between the total ICOHP and Δ*G*_H^*^_ gave a new direction for the design of HER catalyst, which will accelerate the screening process.

## CONCLUSION

Ultra-small hollow ternary alloy nanoparticles (PtNiCu, PtCoCu and CuNiCo) were synthesized for the first time using a simple one-pot method. During synthesis, the displacement reaction and oxidative etching played important roles in the formation of hollow structures. Hollow PtNiCu nanoparticles with a relatively low Pt content showed the highest electrocatalytic activity and stability in an alkaline solution. The overpotential of hollow PtNiCu nanoparticles at 10 mA cm^−2^ was as low as 28 mV versus RHE, corresponding to a Tafel slope of 52.1 mV per decade, and their mass activity was 5.62-fold greater than that of a commercial Pt/C system. The DFT calculations demonstrated that the Δ*G*_H^*^_ of PtNiCu was much closer to zero than that of PtCu, indicating excellent suitability for HER catalysis and synergistic effects among the three metals. Combining theoretical calculations with experimental data, this work provides a promising strategy for the design and preparation of high-performance HER catalysts.

## METHODS

### Preparation of hollow PtNiCu nanoparticles

In the process of synthesizing PtNiCu nanocrystals, copper(II) acetylacetonate (Cu(acac)_2_, 7.5 mg), nickel(II) acetylacetonate (Ni(acac)_2_, 6.5 mg), platinum(III) acetylacetonate (Pt(acac)_3_, 6 mg) and AA (C_6_H_8_O_6_, 52.8 mg) were added to the 25 mL glass tube containing 5 mL OAm. After the glass tube cover was covered, the mixture was sonicated for 2 h to form a uniform solution. The mixture was heated to 180°C for 30 min and maintained for 3 h in an oil bath. After the reaction, the product was washed six times with hexane/ethanol mixture and collected by centrifugation.

### Preparation of hollow PtCoCu nanoparticles

In the process of synthesizing PtCoCu nanocrystals, copper(II) acetylacetonate (Cu(acac)_2_, 7.5 mg), cobalt(III) acetylacetonate (Co(acac)_3_, 7.5 mg), platinum(III) acetylacetonate (Pt(acac)_3_, 6 mg) and AA (C_6_H_8_O_6_, 52.8 mg) were added to the 25 mL glass tube containing 5 mL OAm. After the glass tube cover was covered, the mixture was sonicated for 2 h to form a uniform solution. The mixture was heated to 180°C for 30 min and maintained for 3 h in an oil bath. After the reaction, the product was washed six times with hexane/ethanol mixture and collected by centrifugation.

### Preparation of PtCu nanoparticles

In the process of synthesizing PtCu nanocrystals, copper(II) acetylacetonate (Cu(acac)_2_, 7.5 mg), platinum(III) acetylacetonate (Pt(acac)_3_, 6 mg) and AA (C_6_H_8_O_6_, 52.8 mg) were added to the 25 mL glass tube containing 5 mL OAm. After the glass tube cover was covered, the mixture was sonicated for 2 h to form a uniform solution. The mixture was heated to 180°C for 30 min and maintained for 3 h in an oil bath. After the reaction, the product was washed six times with hexane/ethanol mixture and collected by centrifugation.

### Preparation of hollow CuCoNi nanoparticles

In the process of synthesizing CuNiCo nanocrystals, copper(II) acetylacetonate (Cu(acac)_2_, 10 mg), cobalt(III) acetylacetonate (Co(acac)_3_, 7 mg), nickel(II) acetylacetonate (Ni(acac)_2_, 5 mg) and AA (C_6_H_8_O_6_, 52.8 mg) were added to the 25 mL glass tube containing 5 mL OAm. After the glass tube cover was covered, the mixture was sonicated for 2 h to form a uniform solution. The mixture was heated to 180°C for 30 min and maintained for 12 h in an oil bath. After the reaction, the product was washed six times with hexane/ethanol mixture and collected by centrifugation.

### Catalytic measurements

A three-electrode cell was used at room temperature to measure the electrochemical performance; the glassy carbon electrode (GCE; diameter 5 mm, area 0.196 cm^−2^), graphite rod and Hg/HgO electrode (1 M KOH, *E*(RHE) = *E*(Hg/HgO) + 0.926 V) were used as working electrode, counter electrode and reference electrode, respectively. Two milligrams of catalyst and 1 mg of carbon black were added to 1 mL of water/ethanol/Nafion (ratio 49 : 50 : 1) mixed solution, and then ultrasonicated for 0.5 h to obtain a uniform catalyst ink. Ten microliters of catalyst solution containing hollow PtNiCu nanoparticles, with 0.1326 mg mL^−1^ Pt measured by ICP were dropped on the GCE surface (Pt loading of 0.00676 mg cm^−2^) and dried at room temperature.

The hollow PtNiCu nanoparticles, commercial Pt/C, hollow PtCoCu nanoparticles, hollow PtCu nanoparticles and Cu nanoparticles were prepared by the aforementioned method. The electrocatalytic hydrogen evolution activities of the catalysts were evaluated by LSV (CHI760E, Shanghai Chenhua Instrument Factory, China) in an N_2_-saturated 1.0 M KOH aqueous solution from −0.5 to 0.2 V versus RHE at a scan rate of 5.0 mV s^−1^. The cyclic curves were measured from 0 to 0.12 V versus RHE at a scan rate of 10–50 mV s^−1^ by CV. The current–time curves were measured at 50 mV versus RHE to characterize the electrochemical stability. The electrochemical impedance spectra were measured at 50 mV versus RHE in the frequency range of 0.1–100 kHz. The EACS values were determined by the CV curves in N_2_-saturated 0.5 M H_2_SO_4_ with a scan rate of 50 mV s^−1^.

### Characterization

TEM, HRTEM, HAADF-STEM and EDS images were characterized by Tecnai F20 with an accelerating voltage of 200 kV. The EDS mapping images were characterized by FEI Titan G2 TEM with a probe corrector at 300 kV. The XRD patterns of samples were characterized by a Bruker D8 Advance X-ray power diffractometer operated at 40 kV and 40 mA with Cu Kα radiation (*λ* = 1.5406 Å). The XPS was carried out by an Escalad5 spectrometer with Mg KR radiation (BE) of 1253.6 eV. The elemental content of the samples was determined by ICP-AES (710-ES, Varian). Pore size distributions and surface areas were estimated by a Micromeritics ASAP 2460 analyzer (USA) at liquid nitrogen temperature (77 K).

### Computational details

DFT calculations were performed using Vienna *Ab initio* Simulation Package (VASP) [54,55] with the generalized gradient approximation parameterized by Perdew, Burke and Ernzerhof for the exchange correlation functional [56]. For simulation, we used a four-layer p (3 × 3) slab of Cu(111) slab and fixed the bottom two layers. We replaced the metal atoms that make up the hollow adsorption site to simulate the alloys of different components. An energy cutoff of 400 eV was used for all calculations, and the Γ-centered *k*-point meshes of 4 × 4 × 1 were used for Brillouin zone integration. The atomic positions were relaxed until the force on each atom was <0.05 eV Å^−1^, and the convergence tolerance of the energy was set to be 10^−5^ eV.

## Supplementary Material

nwaa204_Supplemental_File
