# Supplementary material for: Ultra-small hollow ternary alloy nanoparticles for efficient hydrogen evolution reaction
Source: Natl Sci Rev. 2020 Aug 28;8(7):nwaa204. doi: 10.1093/nsr/nwaa204 (PMC8310760; doi:10.1093/nsr/nwaa204)
Supplement: nwaa204_Supplemental_File [file nwaa204_supplemental_file.doc]

Supplementary Information for

**Ultra-small hollow ternary alloy nanoparticles for efficient hydrogen evolution reaction**

Zhenxing Li,^*,1,†^ Chengcheng Yu,^1,†^ Yikun Kang,^2,†^ Xin Zhang,^1^ Yangyang Wen,^1^ Zhao-Kui Wang,^3^ Chang Ma,^1^ Cong Wang,^4^ Kaiwen Wang,^4^ Xianlin Qu,^4^ Miao He,^1^ Ya-Wen Zhang,^5^ and Weiyu Song^*,2^

^1^State Key Laboratory of Heavy Oil Processing, College of New Energy and Materials, Beijing Key Laboratory of Biogas Upgrading Utilization, China University of Petroleum (Beijing), Beijing 102249, China.

^2^College of Science, China University of Petroleum (Beijing), Beijing, 102249, China.

^3^Jiangsu Key Laboratory for Carbon-Based Functional Materials and Devices, Institute of Functional Nano and Soft Mate-rials (FUNSOM), Soochow University, Suzhou, Jiangsu 215123, China.

^4^Beijing Key Laboratory and Institute of Microstructure and Property of Advanced Materials, Beijing University of Technology, Beijing 100124, China

^5^Beijing National Laboratory for Molecular Sciences, State Key Laboratory of Rare Earth Materials Chemistry and Applications, PKU-HKU Joint Laboratory in Rare Earth Materials and Bioinorganic Chemistry, College of Chemistry and Molecular Engineering, Peking University, Beijing 100871, China

Corresponding authors e-mail: lizx@cup.edu.cn; songwy@cup.edu.cn

^†^Equally contributed to this work.


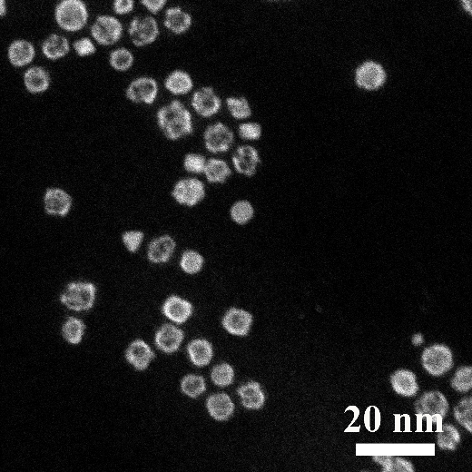


**Supplementary Figure 1.** HAADF-STEM image of hollow PtNiCu nanoparticles.


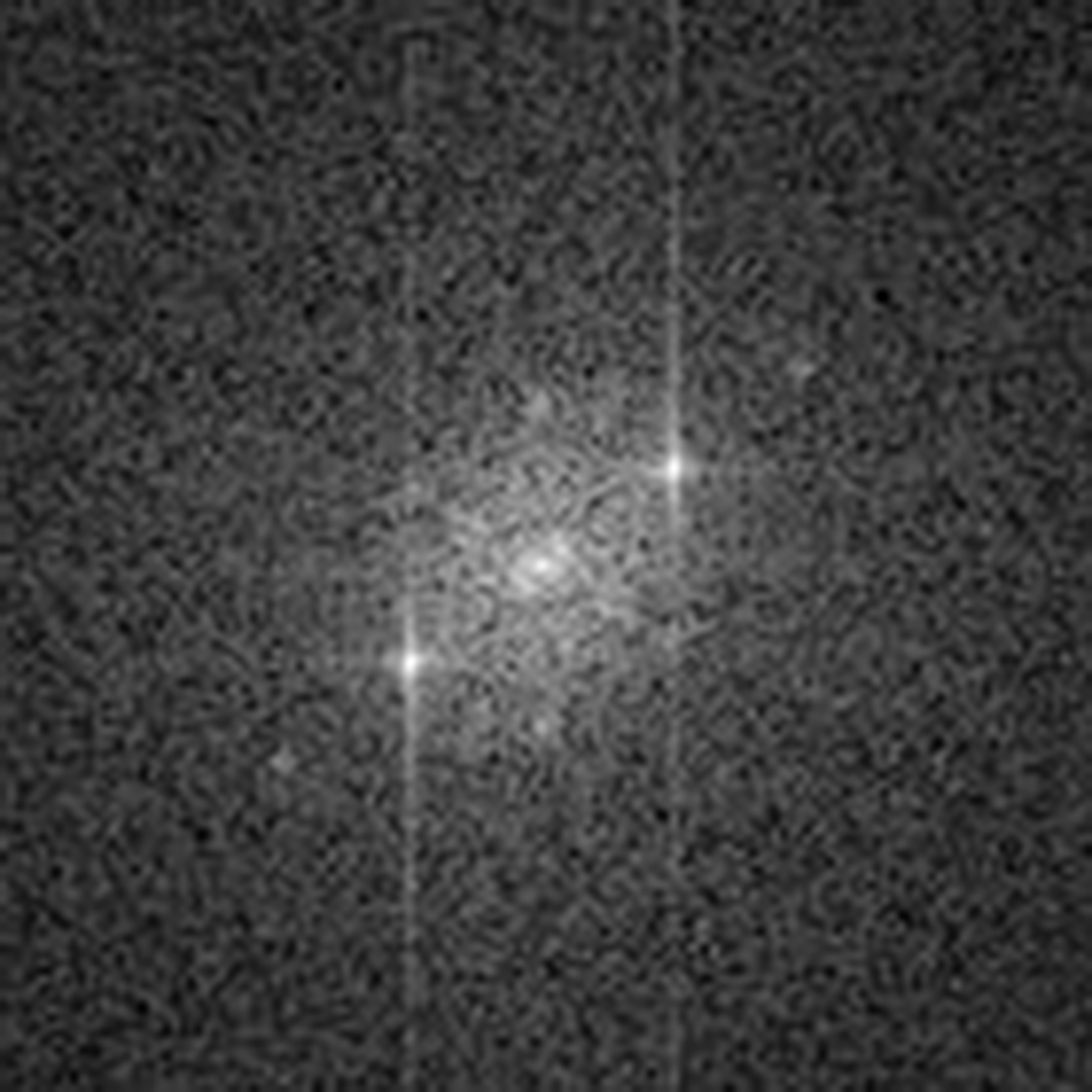


**Supplementary Figure 2.** FFT pattern of hollow PtNiCu nanoparticle.


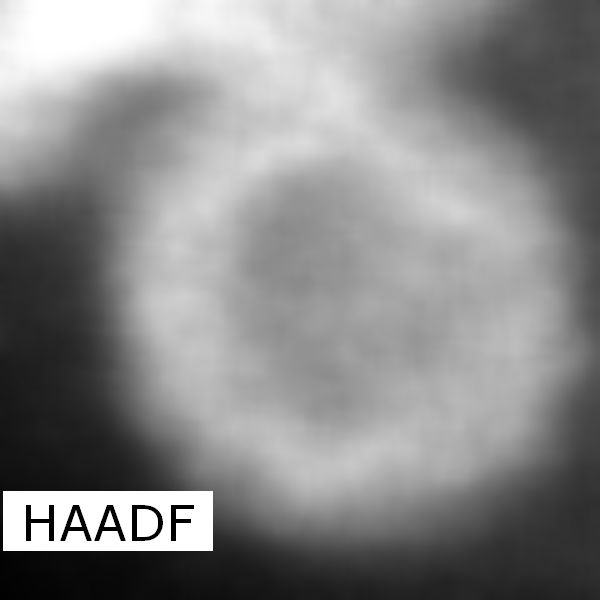


**Supplementary Figure 3.** HAADF-STEM image of a single hollow PtNiCu nanoparticle.


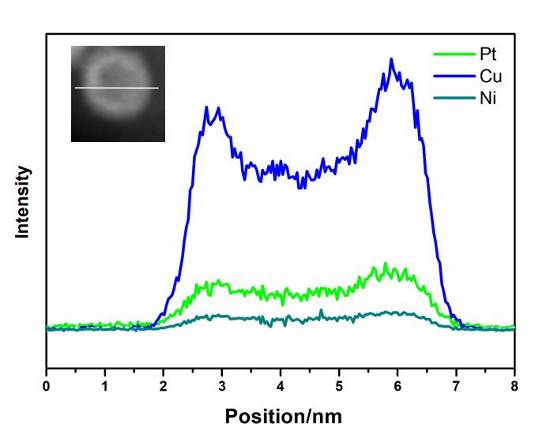


**Supplementary Figure 4.** EDX line scans of hollow PtNiCu nanoparticles.


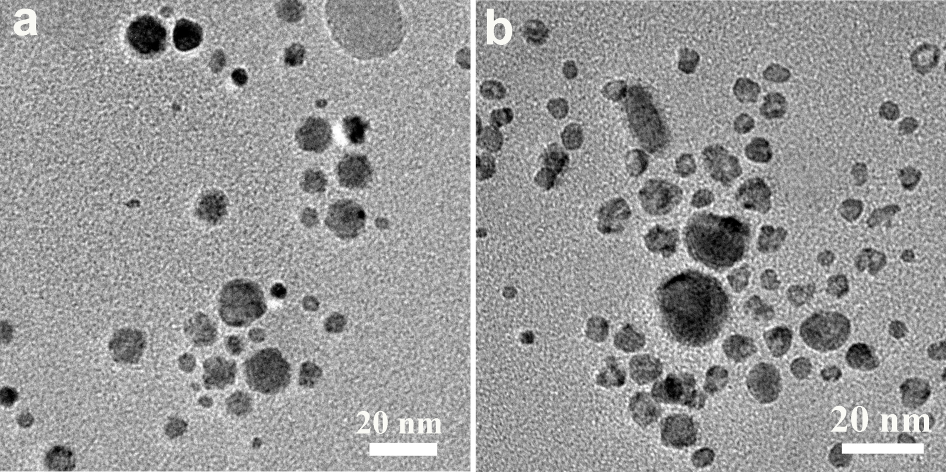


**Supplementary Figure 5.** TEM images of products with the same reaction conditions as that of hollow PtNiCu nanoparticles except using different amounts of Cu(acac)_2_. (a) 5 mg and (b) 10 mg.


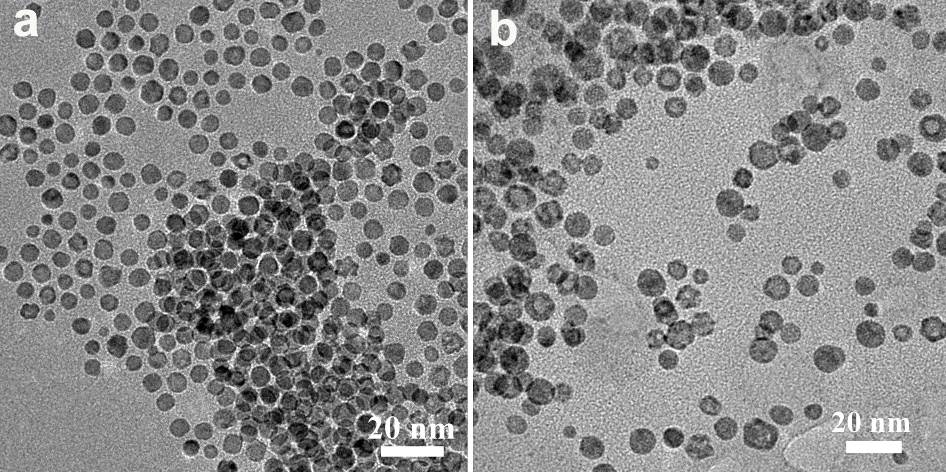


**Supplementary Figure 6.** TEM images of products with the same reaction conditions as that of hollow PtNiCu nanoparticles except using different amounts of AA. (a) 25 mg and (b) 100 mg.


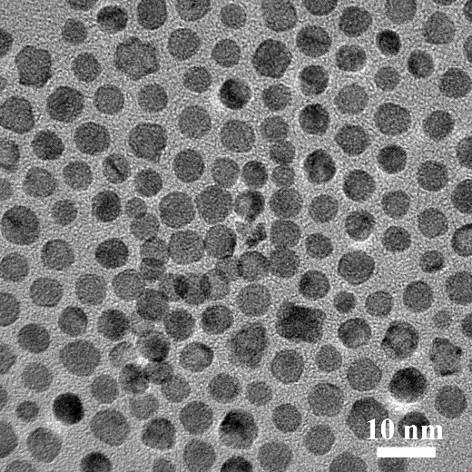


**Supplementary Figure 7.** TEM image of PtNiCu nanoparticles under N_2_ atmospheres.

**
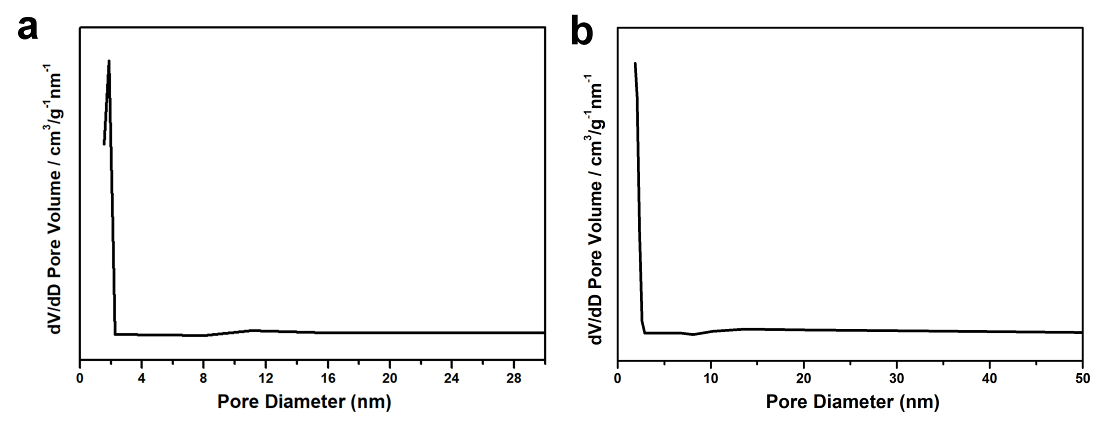
**

**Supplementary Figure 8.** The pore size distribution diagram of (a) hollow PtNiCu nanoparticles and (b) PtNiCu nanoparticles.


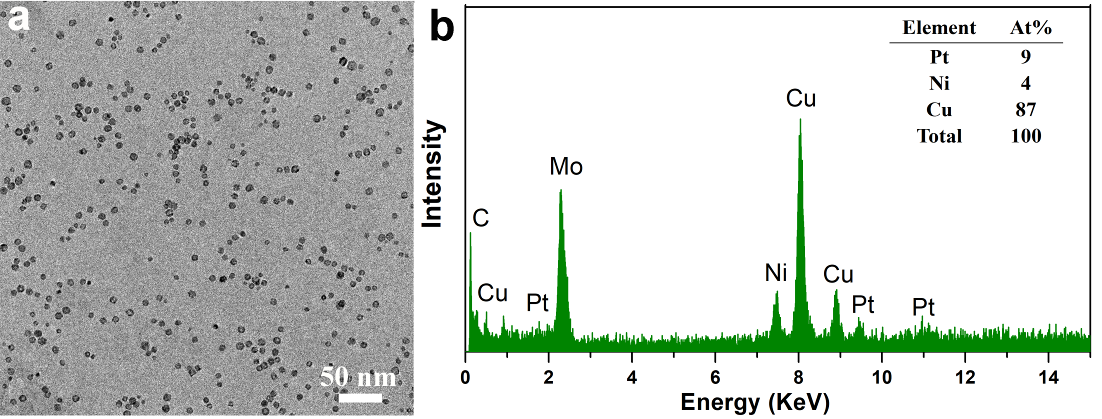


**Supplementary Figure 9.** (a) TEM and (b) EDS of hollow PtNiCu nanoparticles collected from 240 min. Atomic ratio of Pt/Ni/Cu is 9:4:87.


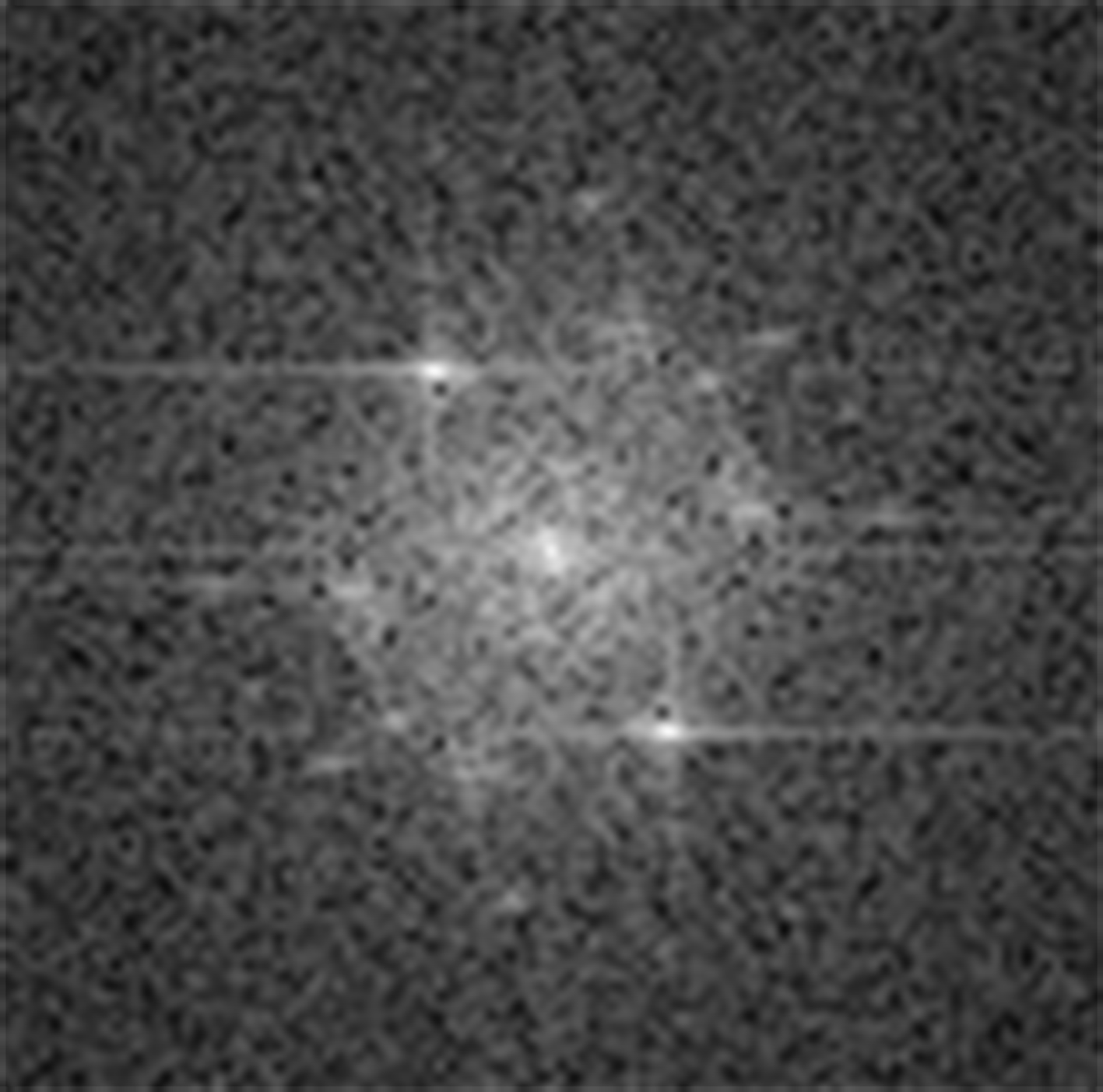


**Supplementary Figure 10.** FFT pattern of hollow PtCoCu nanoparticle.


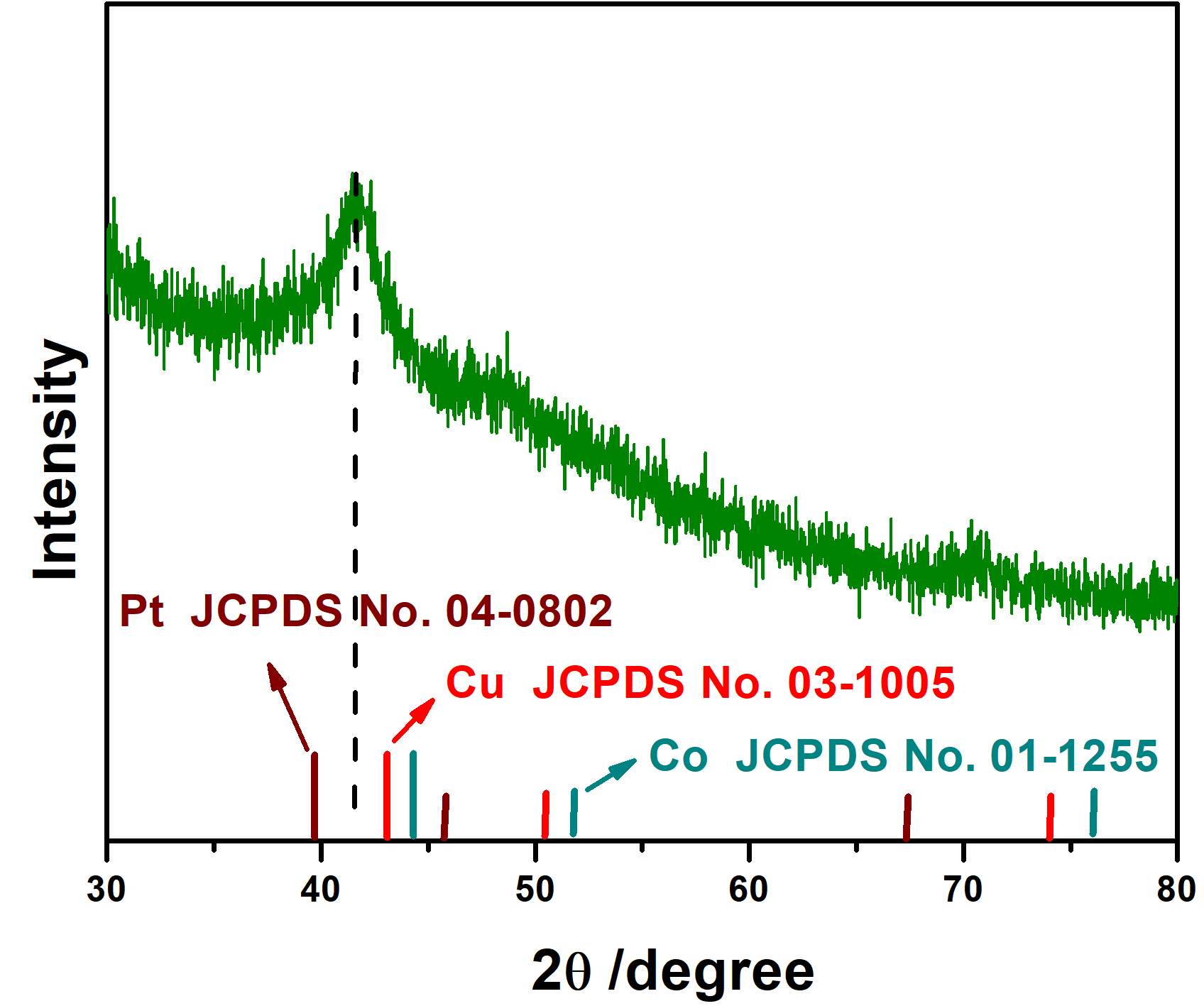


**Supplementary Figure 11.** XRD of hollow PtCoCu nanoparticles.


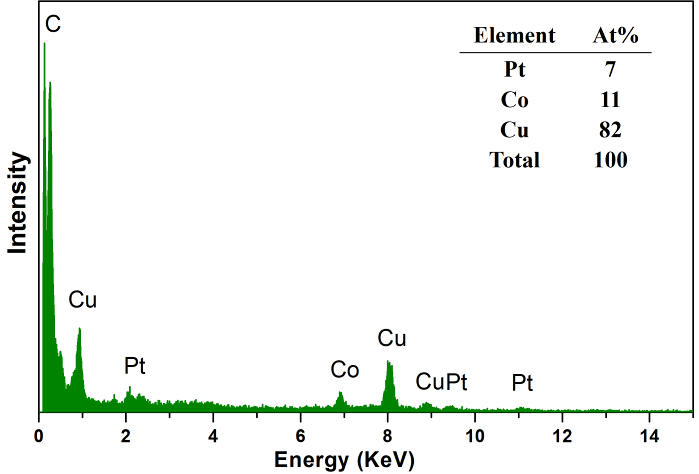


**Supplementary Figure 12.** EDX of hollow PtCoCu nanoparticles.


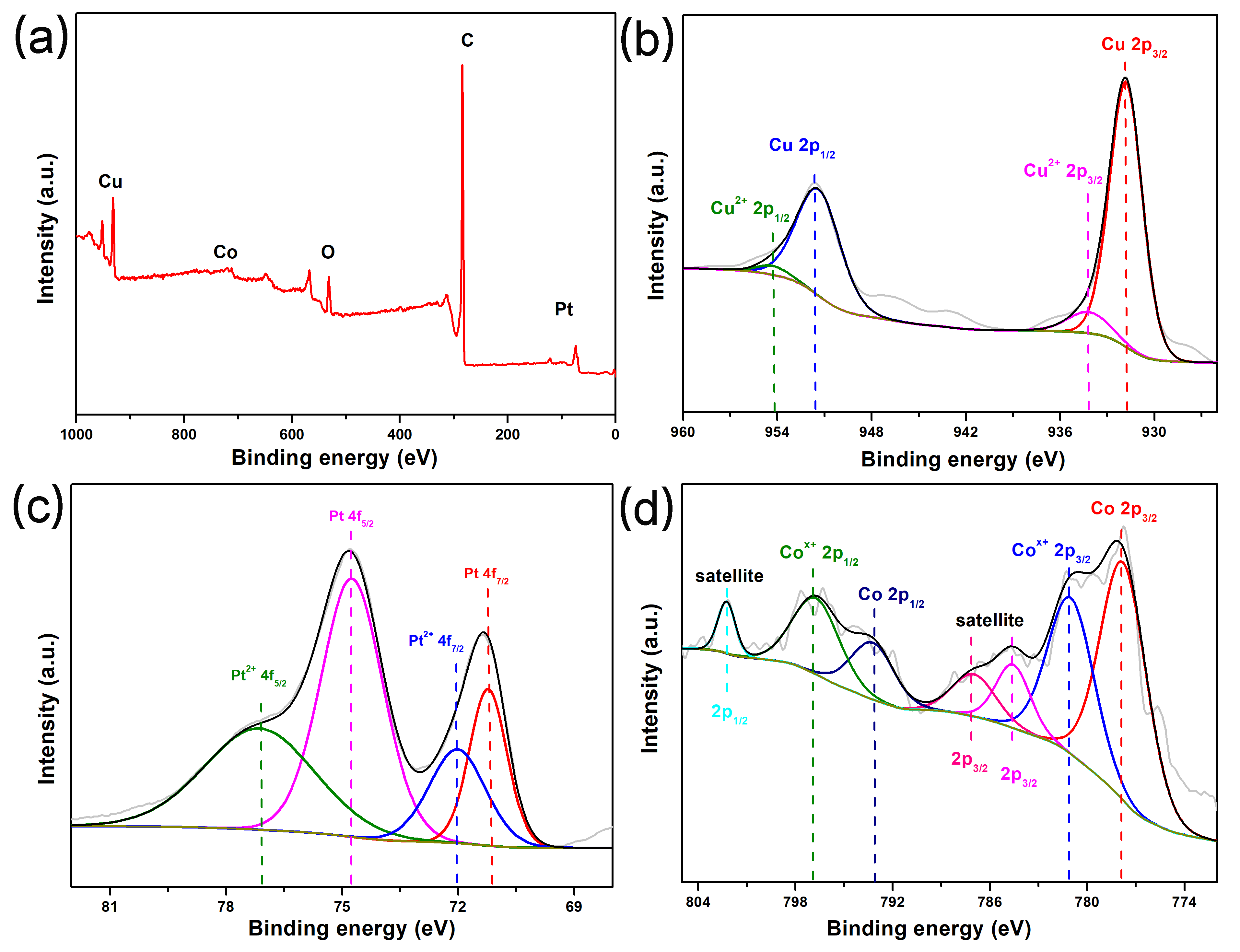


**Supplementary Figure 13.** (a) XPS survey spectrum of hollow PtCoCu nanoparticle. (b) Cu 2p XPS spectrum and (c) Pt 4f XPS spectrum and (d) Ni 2p XPS spectrum of hollow PtCoCu nanoparticle.


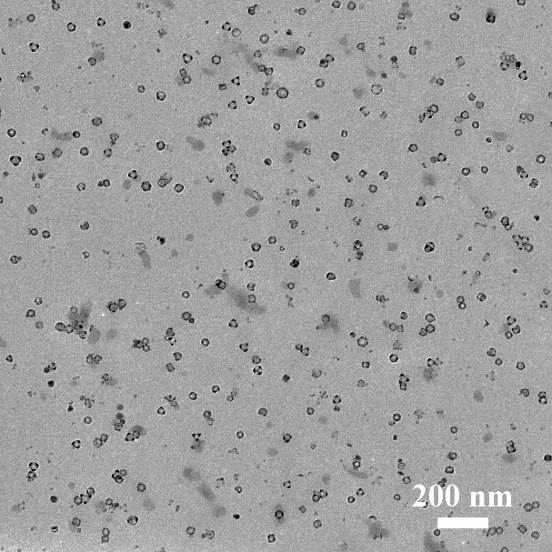


**Supplementary Figure 14.** TEM image of hollow CuCoNi nanoparticles.


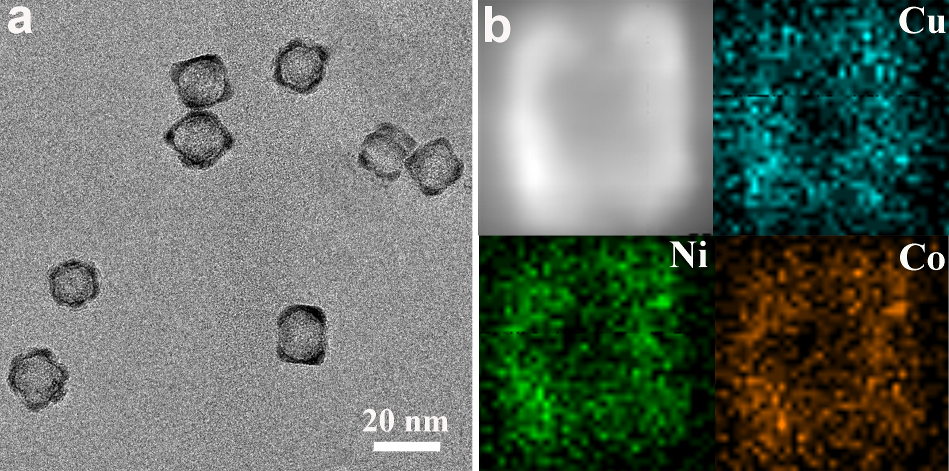


**Supplementary Figure 15.** (a) TEM image of hollow CuCoNi nanoparticles. (b) EDX elemental mappings of hollow CoCuNi nanoparticles.


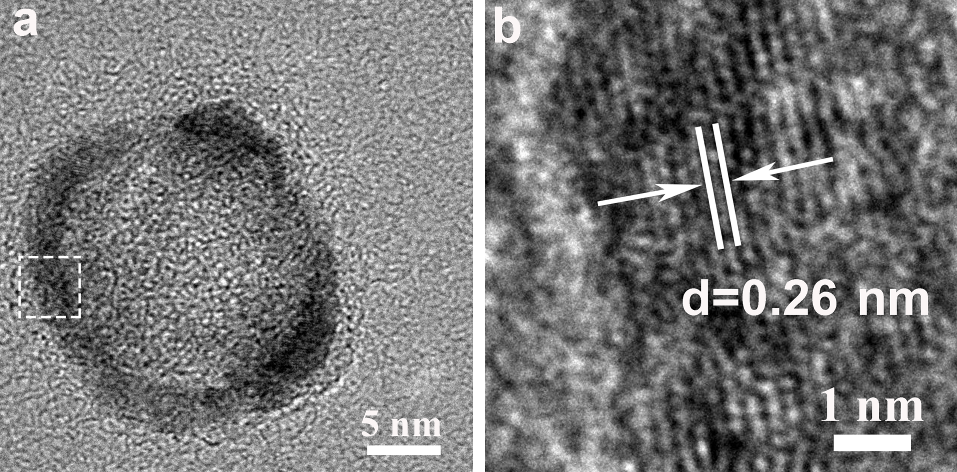


**Supplementary Figure 16.** (a) TEM of sigale hollow CuCoNi nanoparticle. (b) HRTEM of hollow CuCoNi nanoparticles.


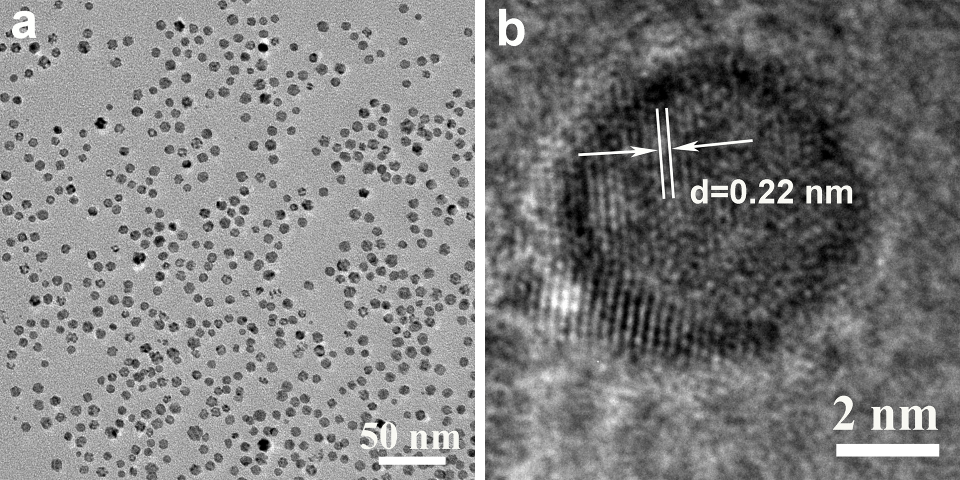


**Supplementary Figure 17.** (a) TEM and (b) HRTEM of hollow PtCu nanoparticles.


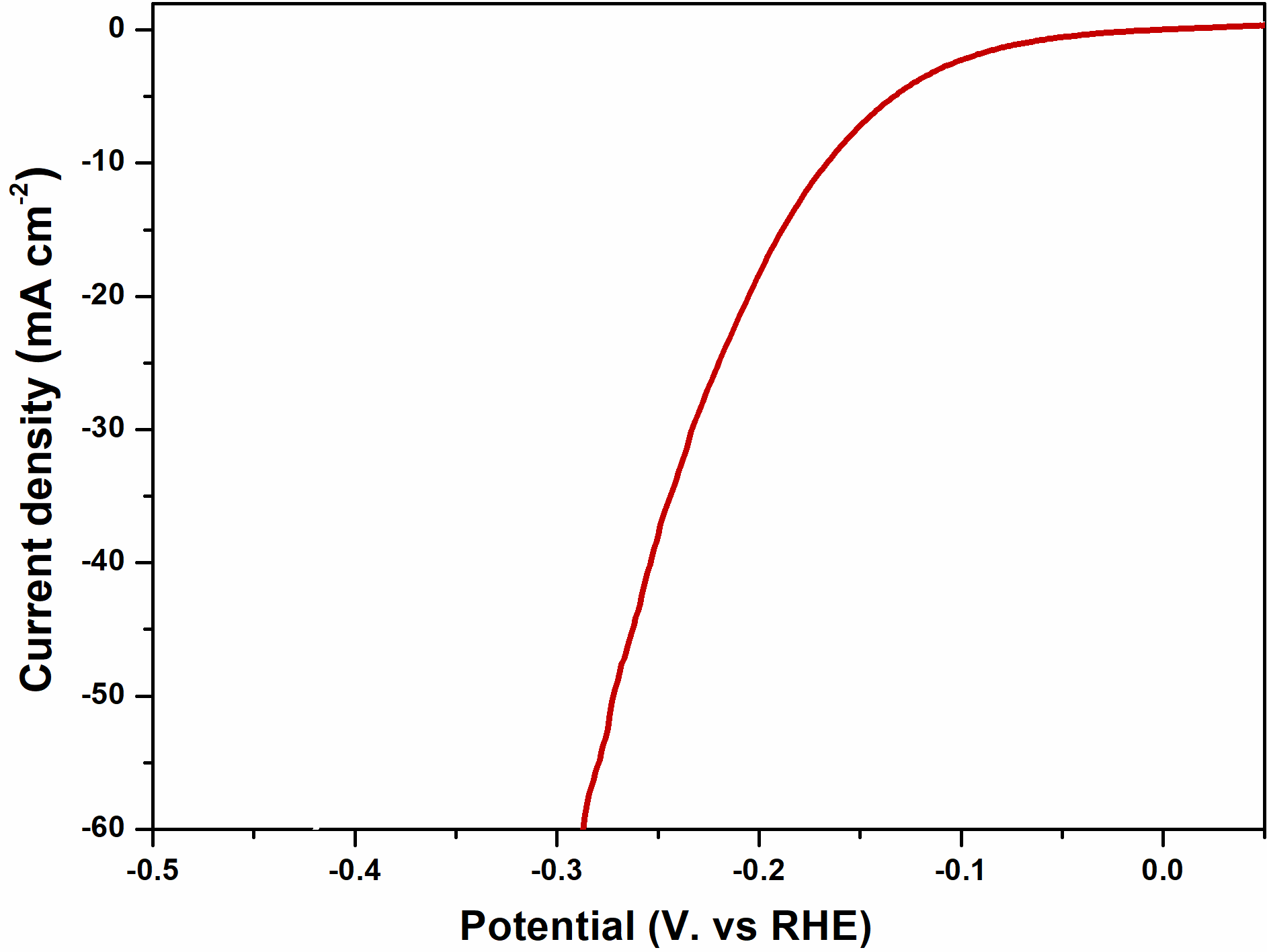


**Supplementary Figure 18.** The polarization curves of hollow CuCoNi nanoparticles.


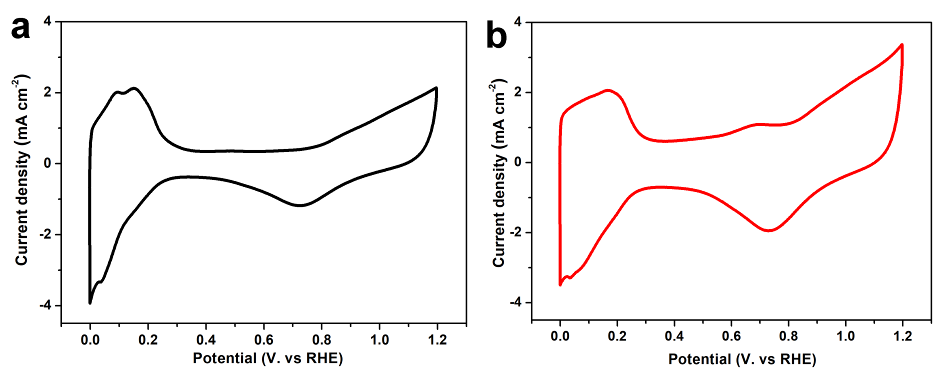


**Supplementary Figure 19.** Cyclic voltammetry curves of (a) hollow PtNiCu nanoparticles and (b) Pt/C in N_2_-saturated 0.5 M H_2_SO_4_ with a scan rate of 50 mV s^-1^.


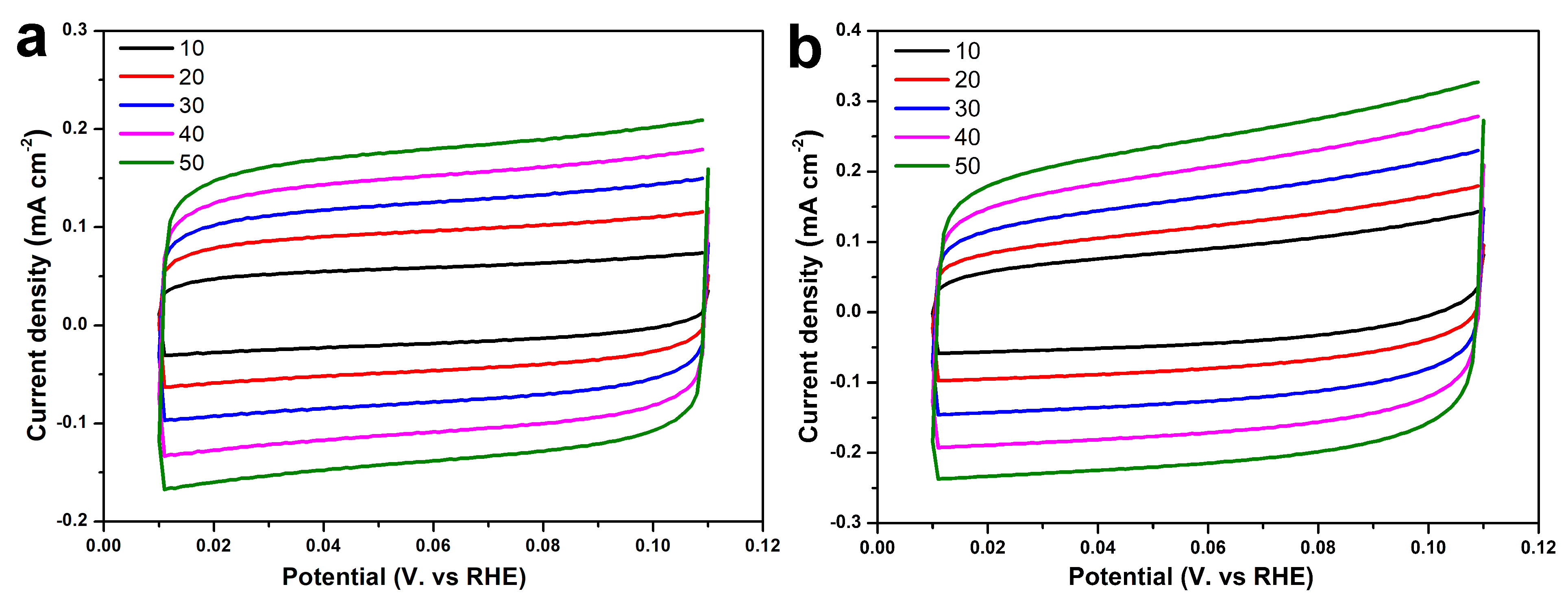


**Supplementary Figure 20.** CV curves of (a) Pt/C and (b) hollow PtNiCu nanoparticle in 1.0 M KOH solution at different scan rates.


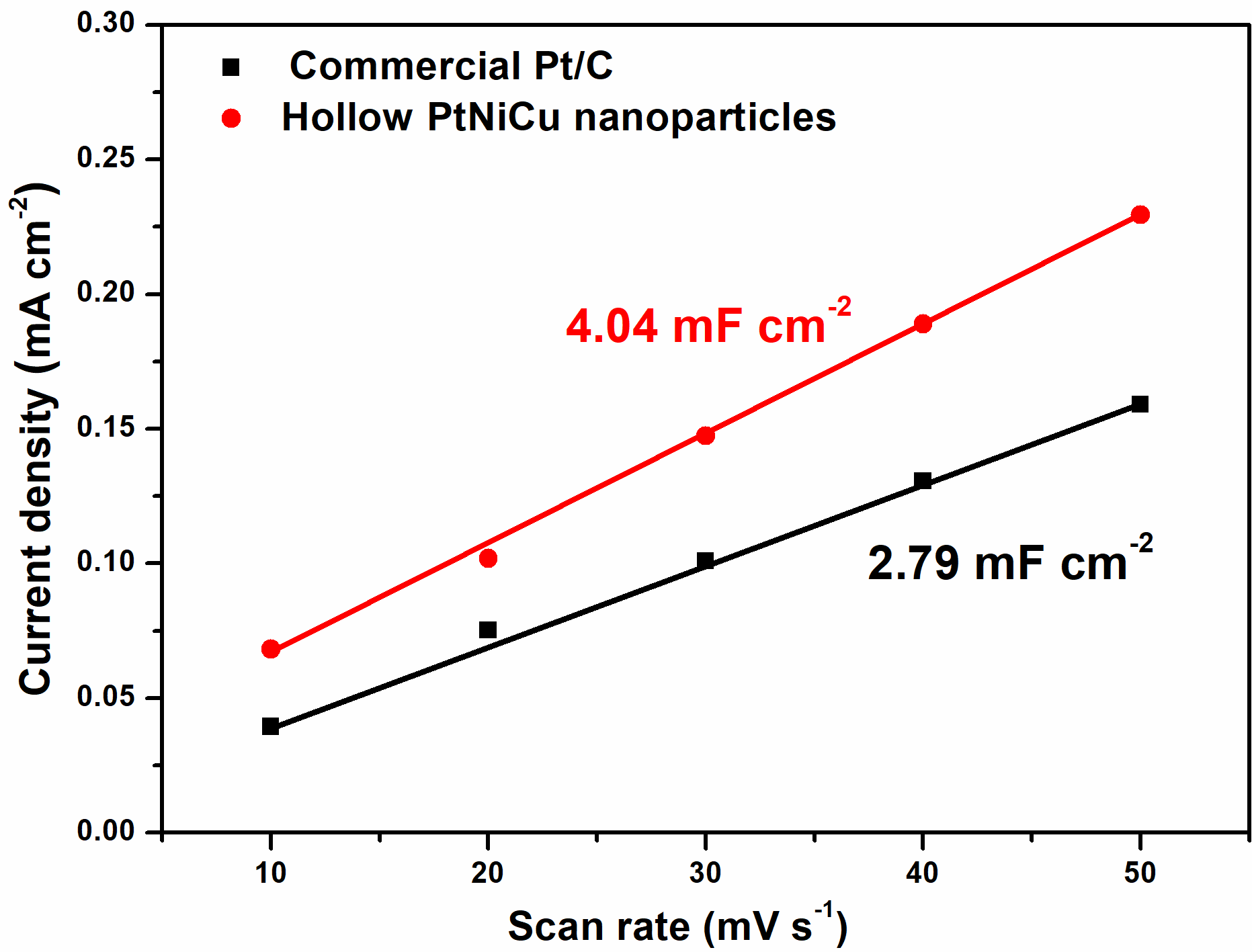


**Supplementary Figure 21.** Linear ﬁtting for the capacitive currents of Pt/C and hollow PtNiCu nanoparticle.


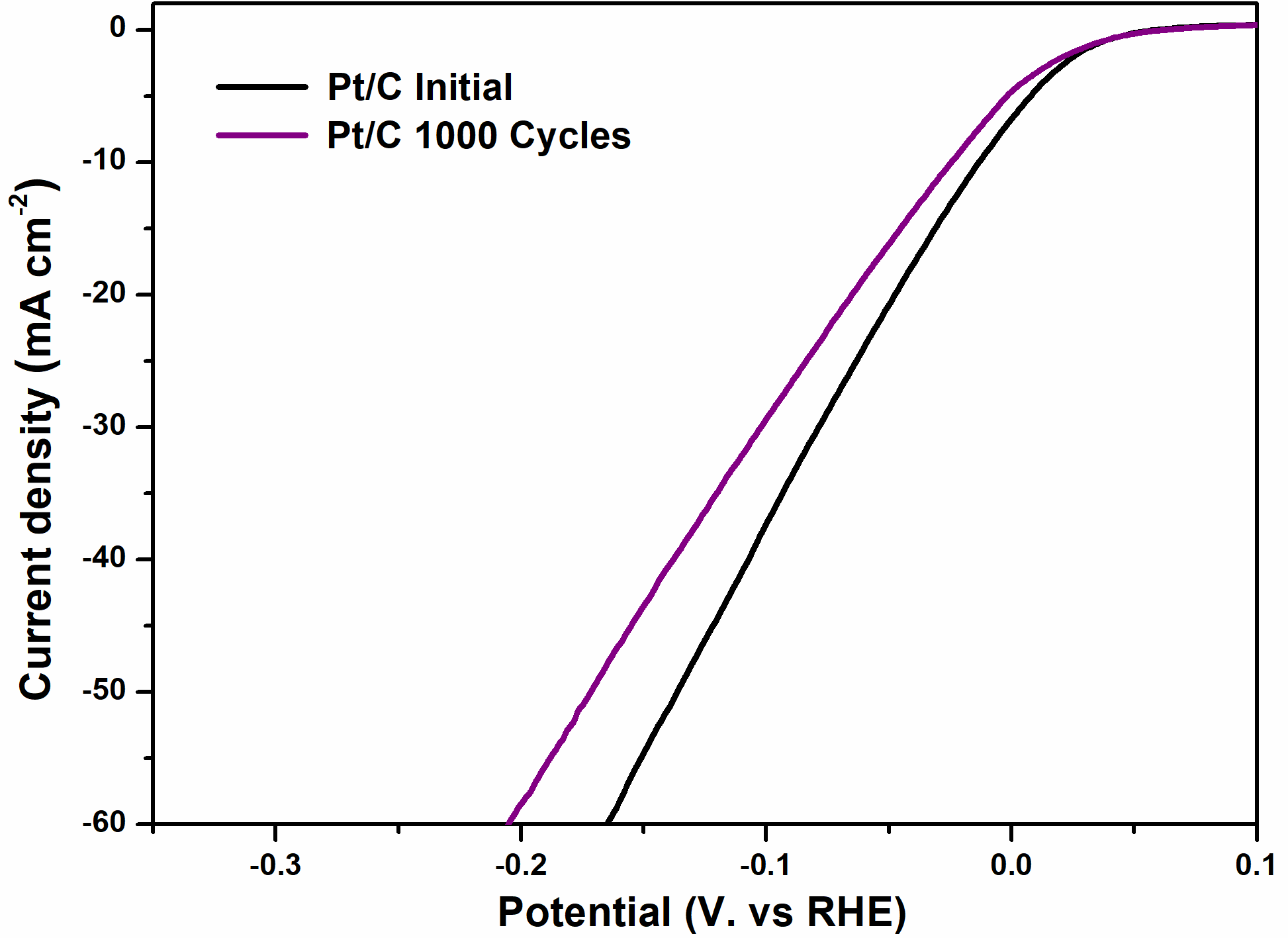


**Supplementary Figure 22.** Cycling stability of Pt/C in 1.0 M KOH solution.


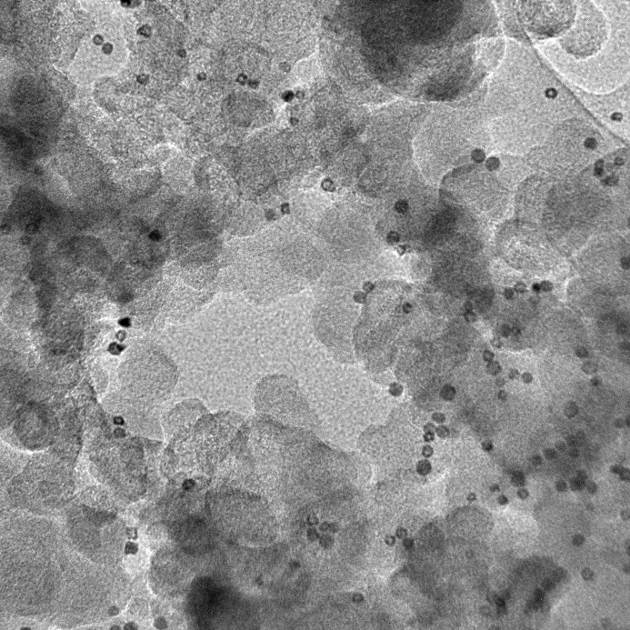


**Supplementary Figure 23.** The TEM of hollow PtNiCu nanoparticles after the electrocatalytic stability test.


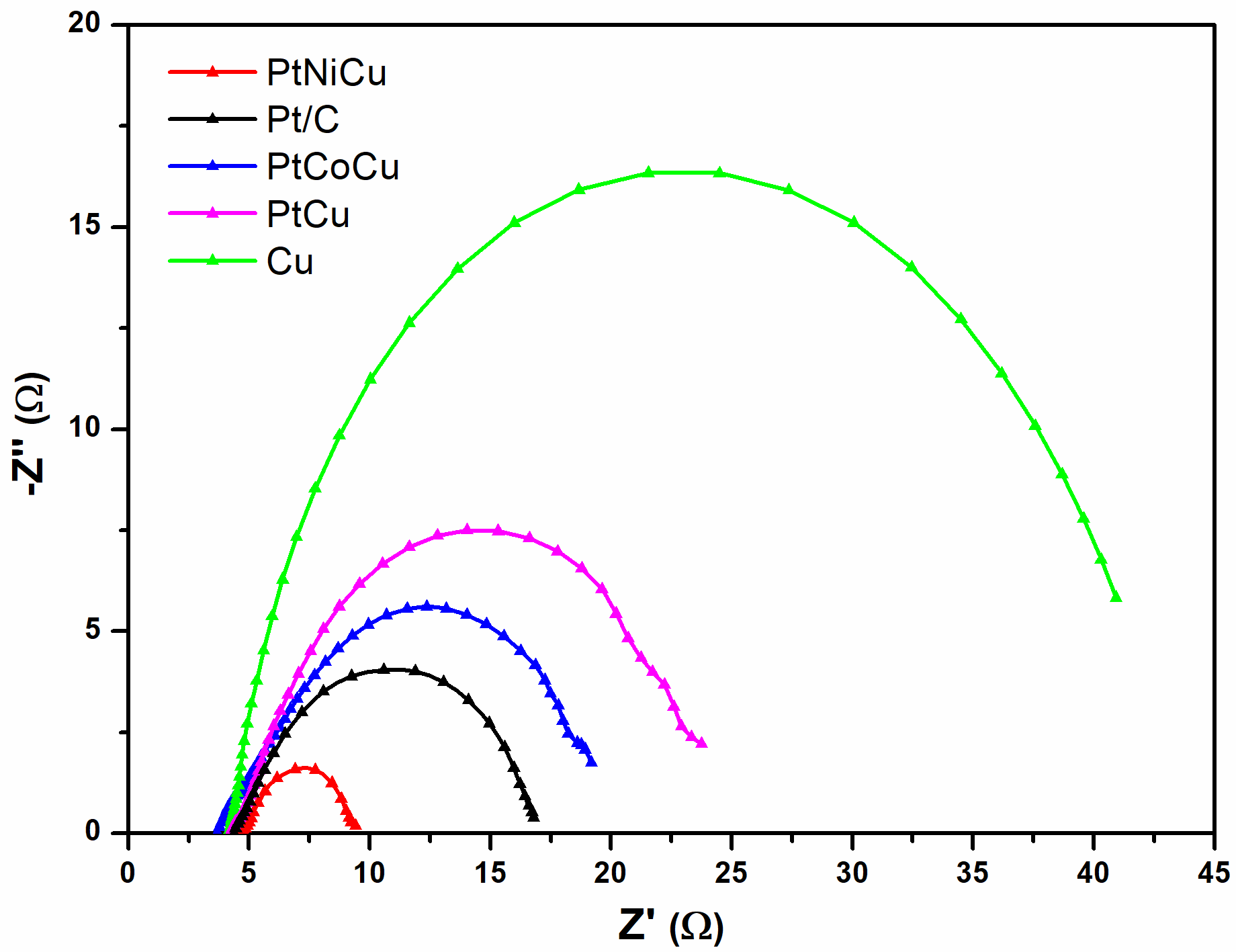


**Supplementary Figure 24.** Nyquist plots of hollow PtNiCu nanoparticles, hollow PtCu nanoparticles, hollow PtCoCu nanoparticles, Cu nanoparticles and Pt/C.


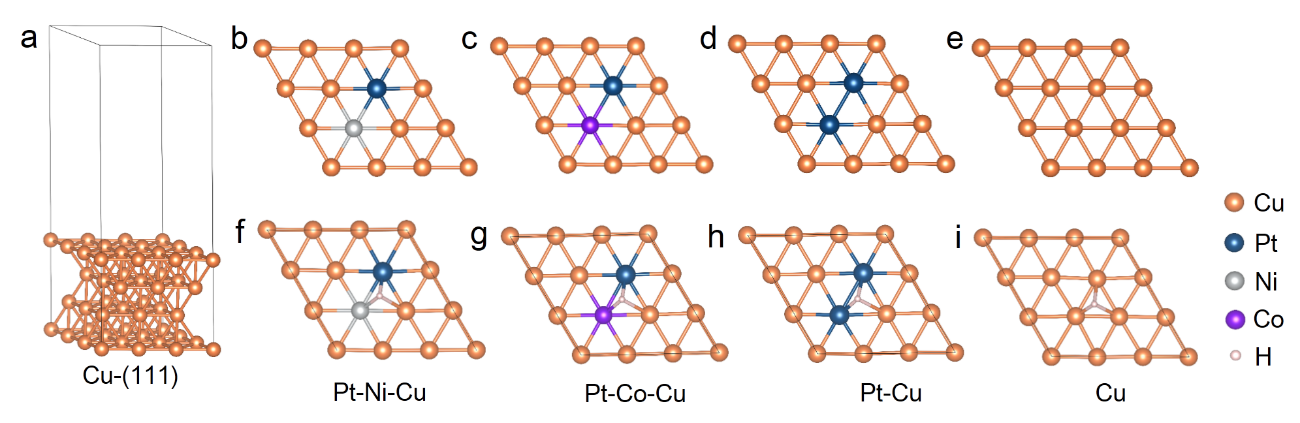


**Supplementary Figure 25.** (a) The optimized slab models for Cu (111). (b)-(e) The models of Pt-Ni-Cu, Pt-Co-Cu, Pt-Cu, and Cu respectively corresponding to the prepared PtNiCu, PtCoCu, PtCu and Cu nanoparticle with hollow structure. (f)-(i) The optimized models of hydrogen adsorption on the slab investigated. For a clear view, only the top layer is shown in (b)-(f).

To determine the Pt-Cu model, we calculated the doping energy (ΔEdop) for different cases. ΔEdop is calculated as follows (n is the number of doped atoms):

ΔEdop = E(doped slab) + nE(Cu atom) - E(Cu slab) - nE(doping atom)


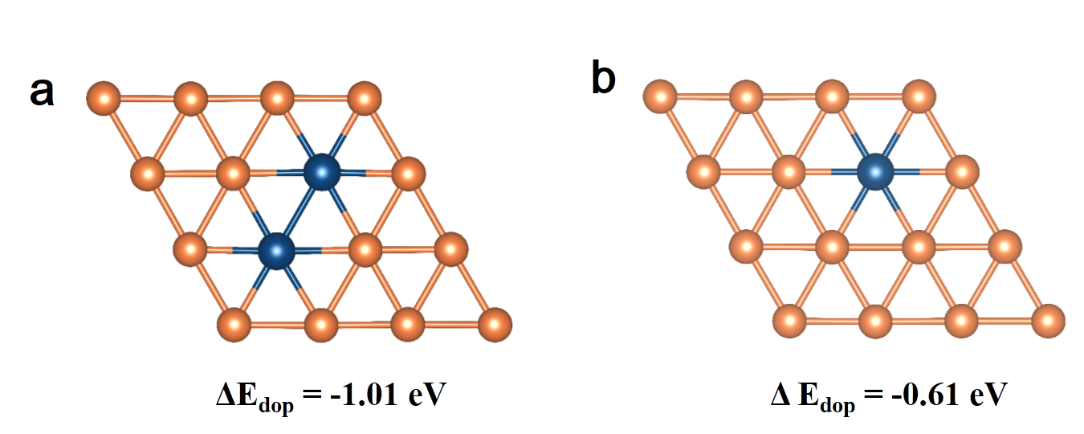


**Supplementary Figure 26.** Possible cases of Pt doped Cu (111) (only the top layer is shown).


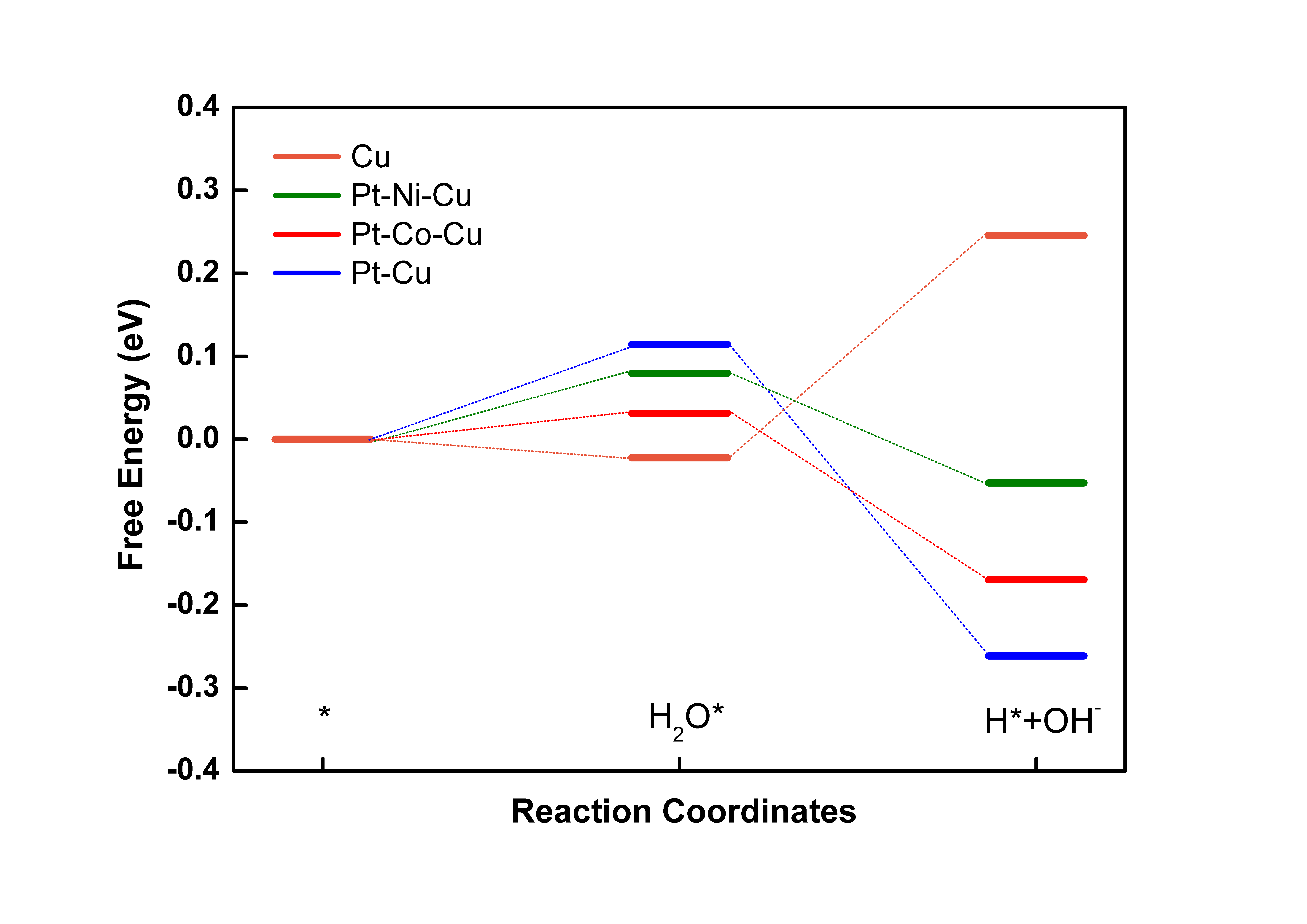


**Supplementary Figure 27.** Free energy diagram of water adsorption and dissociation.

The HER performances were evaluated by computing the reaction free energy of hydrogen adsorption (ΔG_H*_) based on the computational hydrogen electrode model proposed by Nørskov. The free energies of the intermediates were obtained by ΔG_H*_ = ΔE_H*_ +ΔZPE-TΔS, where ΔS and ΔZPE is the entropy change of adsorption hydrogen at 298.15K and zero point energy change respectively (Table S1).

**Supplementary Table 1.** Entropic Energy Contributions (T = 298.15 K) and ZPE Corrections for H_2_ and adsorbed hydrogen.

|  | TS (eV) | ZPE (eV) |
| --- | --- | --- |
| H_2_ | 0.40 | 0.27 |
| H* | 0 | 0.10 |


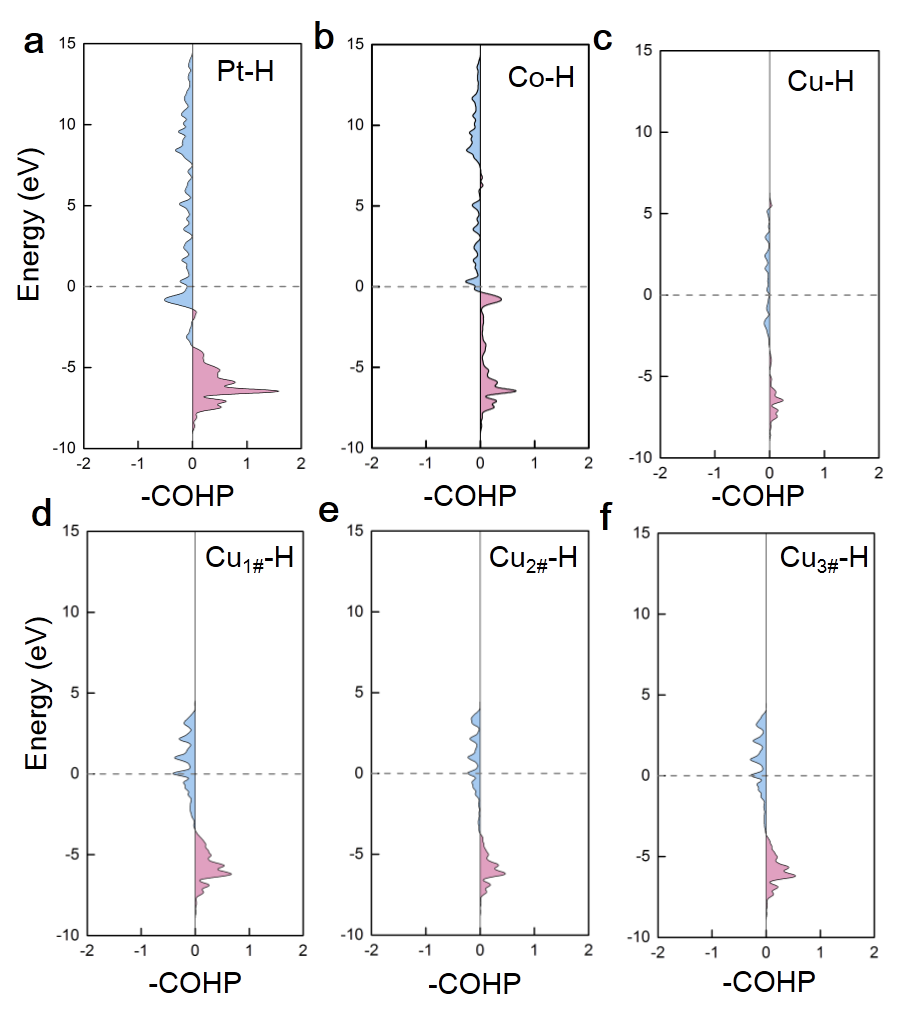


**Supplementary Figure 28.** Projected crystal orbital Hamilton population (pCOHP) for (a) Pt-H, (b) Co-H and (c) Cu-H on Pt-Co-Cu model at hollow adsorption site, and for (d) Cu1#-H, (e) Cu2#-H and (f) Cu3#-H on pure Cu model, respectively.

**Supplementary Table 2.** The elemental ratio of hollow PtNiCu nanoparticle, hollow PtNiCu nanoparticle and hollow PtNiCu nanoparticle testing by XPS.

| Samples | Pt | Cu | Ni/Co |
| --- | --- | --- | --- |
| Hollow PtNiCu nanoparticle | 10% | 85% | 5% |
| Hollow PtCoCu nanoparticle | 9% | 83% | 8% |

**Supplementary Table 3.** The integrated COHP (ICOHP) on Pt-Ni-Cu and their summation (Total ICOHP).

| Pt-H | Ni-H | Cu-H | Total ICOHP |
| --- | --- | --- | --- |
| -1.64 | -0.74 | -0.17 | -2.55 |

**Supplementary Table 4.** ICOHP on Pt-Co-Cu and their summation.

| Pt-H | Co-H | Cu-H | Total ICOHP |
| --- | --- | --- | --- |
| -1.57 | -1.12 | -0.18 | -2.87 |

**Supplementary Table 5.** ICOHP on Pt-Cu and their summation.

| Pt_1#_-H | Pt_2#_-H | Cu-H | Total ICOHP |
| --- | --- | --- | --- |
| -1.37 | -1.75 | -0.14 | -3.26 |

**Supplementary Table 6.** ICOHP on Cu and their summation.

| Cu_1#_-H | Cu_2#_-H | Cu_3#_-H | Total ICOHP |
| --- | --- | --- | --- |
| -0.66 | -0.41 | -0.52 | -1.59 |

**Supplementary Table 7.** Comparison of the HER performance of hollow PtNiCu nanoparticles with other reported HER electrocatalysts in basic electrolyte.

| **Catalyst** | **Tafel slope**  **(mV dec^-1^)** | **Overpotential at 10 mA cm^-2^ (mV)** | **Ref.** |
| --- | --- | --- | --- |
| Pt-Ni/C | - | 60 | 1 |
| Pt_3_Ni_2_-NWs-S/C | - | 42 | 2 |
| PtNi alloy nanomultipods | 78 | 70 | 3 |
| Pt_3_Ni frames/Ni(OH)_2_/C | - | 60 | 4 |
| NiO_x_/Pt_3_Ni Pt_3_Ni_3_-NWs | - | 40 | 5 |
| Pt NWs/SL-Ni(OH)_2_ | - | 85.5 | 6 |
| Ru/C_3_N_4_/C | 69 | 79 | 7 |
| Co(OH)_2_/Pt(111) | - | 248 | 8 |
| PtNi-O/C | - | 39.8 | 9 |
| SANi-PtNWs | 60.3 | 70 | 10 |
| Hollow PtNiCu | 52.1 | 28 | This work |

**References:**

1. Kavian R, Choi S-I and Park J *et al.* Pt-Ni octahedral nanocrystals as a class of highly active electrocatalysts toward the hydrogen evolution reaction in an alkaline electrolyte. *J Mater Chem A* 2016; **4**: 12392.

2. Wang P, Zhang X and Zhang J *et al.* Precise tuning in platinum-nickel/nickel sulfide inter-face nanowires for synergistic hydrogen evolution catalysis. *Nat Commun.* 2017; **8**: 14580.

3. Cao Z, Chen Q and Zhang J *et al.* Platinum-nickel alloy excavated nano-multipods with hexagonal close-packed structure and superior activity towards hydrogen evolution reaction. *Nat Commun* 2017; **8**: 15131.

4. Chen C, Yijin K and Ziyang H *et al.* Highly crystalline multimetallic nanoframes with three-dimensional electrocatalytic surfaces. *Science* 2014; **343**: 1339.

5. Wang P, Jiang K and Wang G *et al.* Phase and Interface Engineering of Platinum-Nickel Nanowires for Efficient Electrochemical Hydrogen Evolution. *Angew Chem Int Ed* 2016; **55**: 12859.

6. Yin H, Zhao S and Zhao K *et al.* Ultrathin platinum nanowires grown on single-layered nickel hydroxide with high hydrogen evolution activity. *Nat Commun* 2015; **6**: 6430.

7. Zheng Y, Jiao Y and Zhu Y *et al.* High Electrocatalytic Hydrogen Evolution Activity of an Anomalous Ruthenium Catalyst. *J Am Chem Soc* 2016; **138**: 16174.

8. Subbaraman R, Tripkovic D and Chang KC *et al.* Trends in activity for the water electrolyser reactions on 3d M(Ni,Co,Fe,Mn) hydr(oxy)oxide catalysts. *Nat Mater* 2012; **11**: 550.

9. Zhao Z, Liu H and Gao W *et al.* Surface-Engineered PtNi-O Nanostructure with Record-High Performance for Electrocatalytic Hydrogen Evolution Reaction. *J Am Chem Soc* 2018; **140**: 9046.

10. Li, M, Duanmu K and Wan C *et al.* Single-atom tailoring of platinum nanocatalysts for high-performance multifunctional electrocatalysis. *Nat Catal* 2019; **2**: 495.
